# Host-Plant Species Conservatism and Ecology of a Parasitoid Fig Wasp Genus (Chalcidoidea; Sycoryctinae; *Arachonia*)

**DOI:** 10.1371/journal.pone.0044804

**Published:** 2012-09-10

**Authors:** Michael J. McLeish, Gary Beukman, Simon van Noort, Theresa C. Wossler

**Affiliations:** 1 Plant Geography Laboratory, Xishuangbanna Tropical Botanical Gardens, Chinese Academy and Sciences, Menglun, Mengla, Yunnan Province, China; 2 Department of Botany and Zoology, DST-NRF Centre of Excellence for Invasion Biology, Stellenbosch University, Matieland, South Africa; 3 Natural History Department, Iziko South African Museum, Cape Town, South Africa; 4 Department of Zoology, University of Cape Town, Rondebosch, Cape Town, South Africa; University of Western Ontario, Canada

## Abstract

Parasitoid diversity in terrestrial ecosystems is enormous. However, ecological processes underpinning their evolutionary diversification in association with other trophic groups are still unclear. Specialisation and interdependencies among chalcid wasps that reproduce on *Ficus* presents an opportunity to investigate the ecology of a multi-trophic system that includes parasitoids. Here we estimate the host-plant species specificity of a parasitoid fig wasp genus that attacks the galls of non-pollinating pteromalid and pollinating agaonid fig wasps. We discuss the interactions between parasitoids and the *Ficus* species present in a forest patch of Uganda in context with populations in Southern Africa. Haplotype networks are inferred to examine intraspecific mitochondrial DNA divergences and phylogenetic approaches used to infer putative species relationships. Taxonomic appraisal and putative species delimitation by molecular and morphological techniques are compared. Results demonstrate that a parasitoid fig wasp population is able to reproduce on at least four *Ficus* species present in a patch. This suggests that parasitoid fig wasps have relatively broad host-*Ficus* species ranges compared to fig wasps that oviposit internally. Parasitoid fig wasps did not recruit on all available host plants present in the forest census area and suggests an important ecological consequence in mitigating fitness trade-offs between pollinator and *Ficus* reproduction. The extent to which parasitoid fig wasps exert influence on the pollination mutualism must consider the fitness consequences imposed by the ability to interact with phenotypes of multiple *Ficus* and fig wasps species, but not equally across space and time.

## Introduction

Underlying mechanisms that maintain the coexistence of widely varying phenotypes within conservative trophic interactions are unclear [1]. Specialisation [2], competition [3], host-associated differentiation [4], and trophic cascading [5] have been shown to influence the generation of the enormous diversity of parasitoids (estimated 20% of insects: [6]). Antagonistic interactions by parasitoids could potentially destabilise or facilitate coexistence among populations of a mutualism [7]. Parasitoid host ranges (number of potential host species) are a fundamental property of ecological interactions e.g. [8], and the breadth of parasitoid host ranges has implications for competition and selection among the lineages they specialise on. The host-plant range limits of parasitoid fig wasps are believed to be comparable to the highly specific host species associations of the pollination mutualism [9], [10], but no empirical accounts have been given. Here we estimate the number of fig tree species (Moraceae: *Ficus*) that support reproduction of parasitoid fig wasp population's and discuss implications for the pollination mutualism.

Parasitoids have been shown to indirectly influence plant fitness traits via their interaction with herbivores [11]. Parasitoids fig wasps lay eggs from the outside of the syconium into galled ovules of other fig wasps (primary host), killing their larvae. This should indirectly influence host-plant fitness by mitigating population sizes of the galling species. Differences in the number of *Ficus* species (secondary host) each fig wasp guild is able to reproduce in might also impact selection on one another [9], [10]. Pollinating (Agaonidae) and non-pollinating (Pteromalidae) fig wasps that gall ovules internally to reproduce are essentially seed parasites. In order for *Ficus* to reproduce, a trade-off between the number of ovules galled (which produce wasps that disperse pollen) and those that are not galled (which can be pollinated to produce seeds) must be met [12]. Generally, lower abundances of parasitoids compared to their prey species [13], [14] and spatial patterns of secondary-host-plant distributions [15], [16] are believed to mediate their coexistence. The relative extent of host ranges is an indication of the potential intensity of competitive interactions [13], [17], and species interactions between fig wasp pollinators and non-pollinators have been shown to be broadly dependent on host specificity and host density [18]. However, no empirical estimates of host-range are known for parasitoid fig wasp populations.

The form of specialisation fig wasps direct at host species remains a key question because of the implications for the pollination mutualism [19], [20], [21], [22], [23]. Specialisation among fig wasps and with *Ficus* implies considerable evolutionary history and the relative differences in the strength of species-specificity among them are not fully realised [21], [24], [25]. Pollinating species show the most extreme specificity towards *Ficus* compared to pteromalid fig wasps [17], although some pollinating species have been shown to reproduce in more than one species [24]. Non-pollinating fig wasps that oviposition internally are arguably less specific to the host fig [19], [26] or appear to be as at least as constrained to host as the pollinators [21]. Tests of phylogenetic congruence have been used in the past to infer host-specificity of parasitoid species and indicate broader host-*Ficus* ranges than pollinator and non-pollinator species [21], [25].

Parasitoid fig wasp speciation has been shown to be a function of host-*Ficus* preferences [27]. Parasitoid fig wasp diversity is responsive to ecological opportunity presented by evolutionary diversification of *Ficus* and indicative of host-plant switching [28]. Plant traits such as volatile organic compounds [29], [30], [31] and syconium morphology [32] have been proposed as partly constraining horizontal transfer by fig wasps among *Ficus* species. For example, the specificity of wasp attraction to two closely related *Ficus* species has been shown to be less for parasitoids than pollinators [33]. Parasitoid fig wasps belong to the subfamily Sycoryctinae (Pteromalidae) and different genera often co-occur in the same fig crop, including *Philotrypesis* and *Apocrypta* [34], *Sycoscapter* [35], and *Watshamiella* and *Arachonia* [36]. Observations of the genus *Sycoryctes* have yet to confirm this life history trait, but it is assumed since the behaviour is ubiquitous in the remaining genera. The Sycoryctinae possess extremely long ovipositors for laying eggs inside the fig syconium. Intraspecific variation of the ovipositor length in the sycoryctini [37], [38] and intraspecific morphological variation in fig syconia [39], [40] are also expected to foster phenotype matching that allows reproduction by a population of parasitoid fig wasps on multiple *Ficus* species. Parasitoid fig wasps might use a comparatively wider yet taxonomically constrained spectrum of traits to locate pollinating and non-pollinating fig wasp host species. However, it is not known whether parasitoid specialisation on *Ficus* is characterised by host switching i.e. different populations reproducing separately on different *Ficus* species (divergent selection), or, whether a population uses multiple host species. It is difficult to discriminate between the process of host switching and a broadening of host-range based on species-level phylogenetic inference because the underlying genetic mechanisms and phenotypes determining host preference are not well understood.

In this study we test the hypothesis that a parasitoid population is able to reproduce in syconia of more than one *Ficus* species. We use parsimony-based and probabilistic methods to discriminate between within-species and between-species haplotype divergence and infer a phylogeny including a nuclear marker to validate species relationships. We assess the morphological variation among putative species lineages to compare with genetic delimitation approaches. Near-exhaustive sampling of all individual *Ficus* trees was possible and provided us with an ecological ‘snapshot’ of a fig wasp community located within a patch of primary and mixed forest. Specimens were reared from all trees in the forest that were releasing fig wasps. Variation at the cytochrome oxidase subunit one (*COI*) and cytochrome b (*Cytb*) loci for the most commonly occurring parasitoid genus was compared across *Ficus* species. We tested: i) whether single or multiple populations/species of *Arachonia* were present in Kibale Forest; ii) their relationship with specimens collected widely over Eastern and Southern Africa; and iii) haplotype structuring according to the *Ficus* species they were reared from. Our findings indicate that a parasitoid population of *Arachonia* was able to reproduce in multiple *Ficus* species in the same forest patch in the period concomitant with the developmental time of a single generation.

## Results

### Statistical parsimony and AMOVA

We generated a haplotype network using statistical parsimony to explore *a priori* criteria for discriminating within and between species-level divergences at the *COI* and *Cytb* mtDNA loci. Our *COI* and *Cytb* sequence data were collapsed into 92 unique haplotypes (of 145 specimens) with 10 networks. Six of these were represented by a single haplotype. These singleton taxon networks we generated comprised specimens reared from *F. sycomorus*, *F. umbellata*, *F. ovata*, *F. sansibarica* and *F. sur*. Reticulations were present in the some of the four remaining major networks. Our results demonstrated several lines of evidence that show haplotype structuring is a function of *Ficus* host associations and the geographic region from which the specimens were collected. The levels of genetic structuring we uncovered at Kibale appeared independent of the year in which a few specimens were collected. During the month long census over August in Kibale Forest, 116 individual trees were recorded with 11 of them releasing fig wasps; less than 10% of the fig trees (Table 1). For instance, 1 of 22 *F. chirindensis*, 2 of 28 *F. artocarpoides*, 1 of 2 *F. ovata*, 3 of 30 *F. natalensis* (not releasing *Arachonia*), 1 of 2 *F. polita* (the collection was made from a morpho-type that was near *F. umbellata*), and 1 of 7 *F. sur* within Kibale were releasing fig wasps over the sampling period.

**Table 1:** *Ficus* species of Kibale Forest in Uganda.

Hosts with *Arachonia*	Host with no *Arachonia*	Present but no figs
***Galoglychia***subsection *Caulocarpe*	***Eriosycea***	***Galoglychia***
*F. artocarpoides*	*F. asperifolia*	*F. polita*
*F. chirindensis*		*F. sansibarica* (?)
*F. ovata*	***Galoglychia***	
*Ficus* sp. nov. near *polita/umbellata*	*F. natalensis*	***Galoglychia***
	*F. persicifolia*	*F. conraui*
***Sycomorus***subsection *Sycomorus*		*F. ottoniifolia*
*F. sur*		*F. saussureana*
*F. sycomorus*		
		***Sycomorus***
		*F. mucuso*
		*F. vallis-choudae*
		**Unknown**
		*F. sp.* unknown 1
		*F. sp.* unknown 2
		*F. sp.* unknown 3
		*F. sp.* unknown 4

Species for which *Arachonia* were reared, those species where no *Arachonia* were reared but other fig wasps were, and species that were not producing syconia releasing wasps. *Ficus* section is indicated at the start of each group.

**Figure 1:**
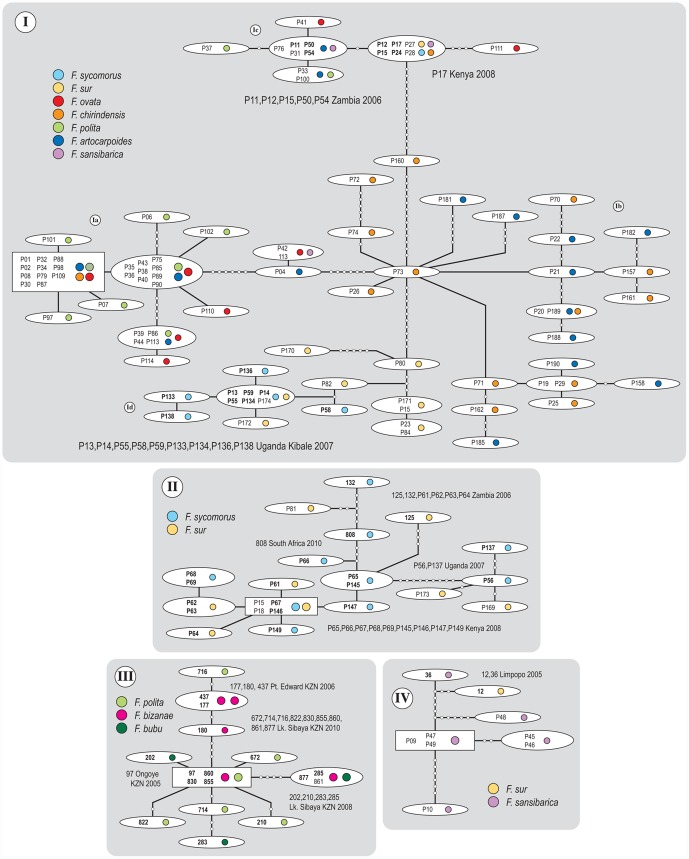
*Arachonia COI* and *Cytb* haplotype networks inferred using statistical parsimony. Small white circles infer 1-step mutations. Specimen codes indicated inside ellipses. Colour circles show *Ficus* species affiliation. All haplotypes except those codes indicated in bold type were collected from Kibale Forest in Uganda August 2008. Circled Roman numerals indicate AMOVA group designations. The lower left network was collected entirely from within KwaZulu Natal (KZN) in South Africa.

**Figure 2:**
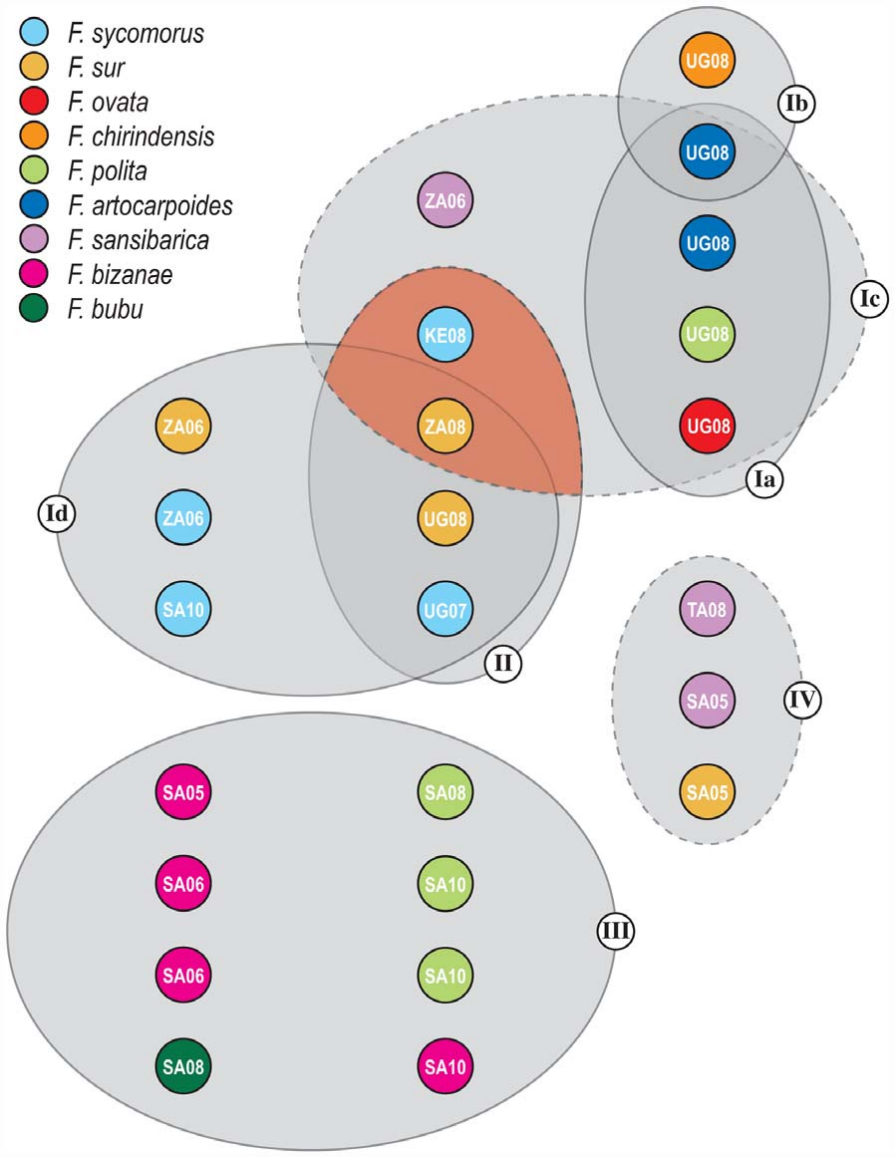
Schematic summary of *Ficus* species that were used by *Arachonia* haplotype groups. Groups circled Ia through IV were inferred using statistical parsimony, AMOVA, and the GMYC test. Grey ellipses' indicate different haplotype groups (dashed outline indicates use of two *Ficus* sections by a single haplotype group). The red-shaded section of overlap indicates a discrepancy with the Bayesian haplotype tree (Figure 3) where the two specimens on section *Sycomorus* (KE08 & ZA08) in group Ic cluster with groups Id and II instead. Abbreviations: UG, Uganda; KE, Kenya, ZA, Zambia; TA, Tanzania; and SA, South Africa (numerals indicate year of collection).

Our statistical parsimony analysis showed some ambiguity in haplotype assignment. This largely occurred because of missing characters at flanking ends of some (55 of the 290 COI & Cytb sequences) fragments. These ambiguous connections might signify homoplasies that cannot be assigned a single connection. We used the procedure recommended by Posada and Crandall in [41] to establish the most plausible connections. Network I comprised the most haplotypes (Figure 1). We coarsely divided network I into four groups (Ia, Ib, Ic, Id) that are more or less separated by the largest number of mutation steps. These groups are intended to simplify extrapolation across analyses rather than taxonomic affiliations. Each of the four groups is clearly identifiable by the *Ficus* species they were reared from. The highest concentration of haplotypes (group Ia) was collected from *F. ovata*, *F. chrinidensis*, *F. polita*, and *F. artocarpoides*. The negligible genetic variance within this group of haplotypes was independent of *Ficus* species affiliation. The next most genetically similar group (Ib) was collected from only two *Ficus* species, *F. chirindensis* and *F. artocarpoides*. Again, the genetic structuring of the haplotypes within this group was independent of the two *Ficus* species this population reproduced on. In other words, our results show no evidence that *Ficus* affiliation of a given population structured the genetic variance of that population. Both haplotype groups Ia and Ib were collected entirely from Kibale Forest during August 2008. The haplotype group Ic included collections from *F. ovata*, *F. artocarpoides*, *F. sansibarica*, *F. polita*, *F. chirindensis*, *F. sycomorus*, and *F. sur* made in Kibale, elsewhere in Uganda, Zambia, and Kenya. This network included one haplotype found on four species (*F. sur*, *F. sycomorus*, *F. chirindensis*, and *F sansibarica*) in Kibale in the 2008 sampling period. Haplotype group Id was collected from *F. sur* and *F. sycomorus*, which are not parasitized by groups Ia and Ib. Group Id included haplotypes from Kibale collected one year earlier and another from Zambia two years earlier and all from *F. sur* and *F. sycomorus*. The four groups (Ia–Id) of haplotypes are arguably distinct populations or putative genetic *Arachonia* species. We show that each taxon has host *Ficus* species ranges >1. The most apparent distinction in *Ficus* range among the groups is evident in the split between the population parasitizing *Ficus* from section *Sycomorus* (subsection *Sycomorus*) and those from section *Galoglychia* (subsection *Caulocarpae*). For instance, haplotype group Id (Figure 1) was affiliated with species of section *Sycomorus*, but closely related to groups on other species (Ia, Ib, Ic). Haplotype group II is distinct and also comprises specimens from across Southern Africa. There is evidence of cross-*Ficus* section species ranges from group Ic. However, our Bayesian haplotype tree inference indicated that the two specimens from group Ic on *F. sycomorus* and *F. sur* cluster with groups Id and II that are all affiliated with these species of section *Sycomorus* (see below). The three remaining networks II–IV (Figure 1) each reflect generally distinct geographical range differences. Network II comprised haplotypes that were reared from *F. sur* and *F. sycomorus* were collected over several years from Kenya, Uganda, Zambia, and one from South Africa. *Arachonia* species shown in network III were collected from *F. polita*, *F. bizanae*, and *F. bubu* in KwaZulu Natal South Africa. Network IV shows a second example of a haplotype group that has included in their range, *Ficus* species from both sections *Sycomorus* and *Galoglychia*. A schematic of *Ficus* species range differences among the haplotype networks is given in Figure 2. There were two individuals of *F. artocarpoides* in Kibale Forest that were receptive to oviposition, but members of different haplotype groups (Ia & Ib) were collected from different individuals.

We conducted analysis of molecular variance that requires *a priori* designation of populations. The four networks (I, II, III, and IV) resulting from our statistical parsimony analyses were nominated as populations and used in the initial AMOVA. A second AMOVA was conducted with network I deconstructed into four populations (Ia, Ib, Ic, and Id) in addition to populations II, III, and IV (Table 2). Our initial AMOVA among four putative populations resulted in an *F_ST_* = 0.52 (*P*<0.001) with variance within and among groups being approximately equivalent (Table 2). The second test among 7 putative populations resulted in an *F_ST_* = 0.90 (*P*<0.001). The variance between and within groups was substantially different and indicative of maximally distinct groups of haplotypes, each associated with different sets of *Ficus* host species.

**Table 2:** AMOVA results for two different group designations.

		d.f.	Sum of Squares	Variance	% Variation
**Among**		3	700.0	8.3	51.9
**Within**		142	1096.7	7.7	48.1
Total		145	1796.7	16.0	
***F_ST_***	0.52 (**P<0.001)**				
**Among**		6	1590.2	12.8	89.6
**Within**		139	206.5	1.5	10.4
Total		145	1796.7	14.3	
***F_ST_***	0.90 **(P<0.001)**				

Networks I, II, III, and IV (above; see Figure 1); and networks Ia, Ib, Ic, Id, II, III, and IV (below; see Figure 1).

### Putative species delimitation

We used a GMYC likelihood test to estimate which haplotype groups best fit either a coalescent or Yule model of branching. Genetic divergences were estimated using the ultrametric consensus phylogeny implemented under a Bayesian approach (Figure S1). The GMYC likelihood test was significant (*P*<0.001). The clustering of lineages representative of the population-level branching model were largely concordant with the networks estimated using statistical parsimony. The mixed model likelihood test identified 13 clusters (CI: 12–17) consistent with population-level branching patterns and 19 entities (CI: 18–26) typical of the species-level branching model. The level of *Ficus* host species conservatism exhibited by *Arachonia* clades specialising on either section *Sycomorus* or section *Galoglychia* evident from the statistical parsimony analysis was concordant with phylogenetic inferences. There were rare exceptions to this within-section conservatism in each of the two major stem clades associated with either section *Sycomorus* (subsection *Sycomorus*) or section *Galoglychia* (subsection *Caulocarpe*).

**Figure 3:**
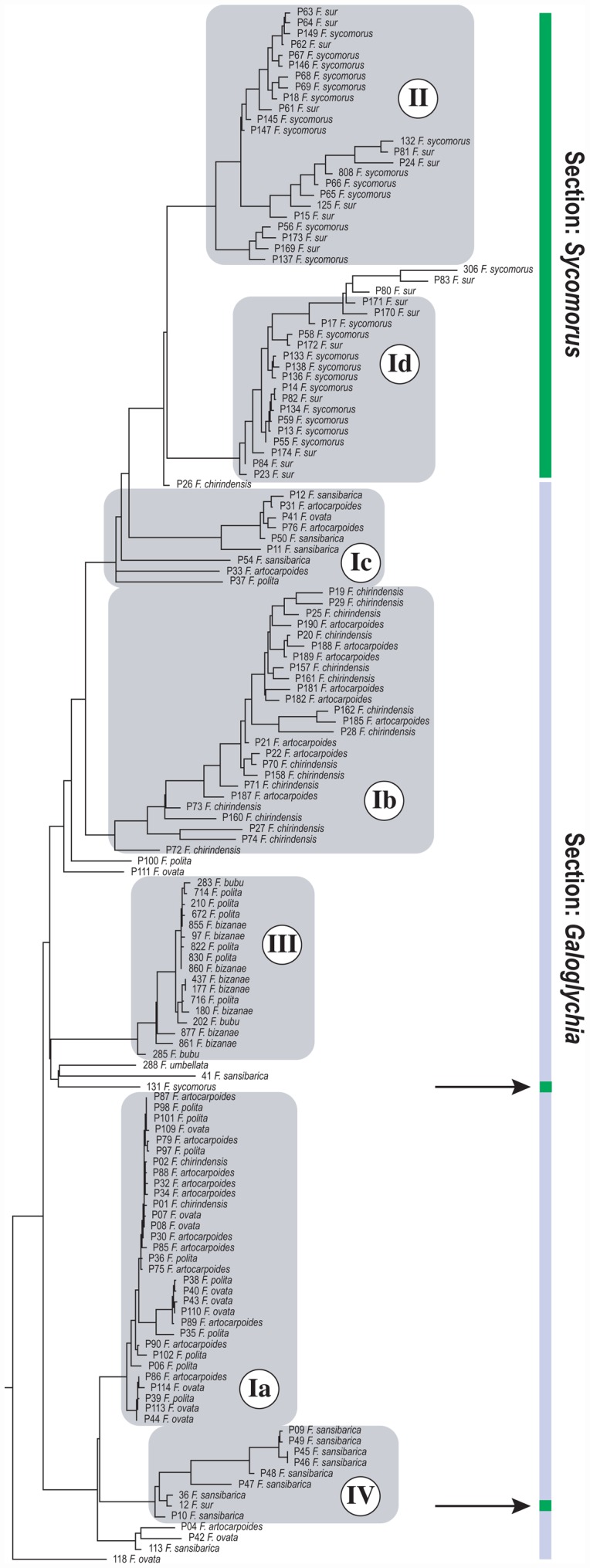
Bayesian consensus phylogram of *Arachonia COI* and *Cytb* haplotpyes. Host *Ficus* section is indicated with vertical coloured bars. Arrows indicate polyphyletic *Ficus* associations with *Sycomorus* within otherwise *Galoglychia-*affiliated lineages. Posterior probability node support is given in Figure S2.

**Figure 4:**
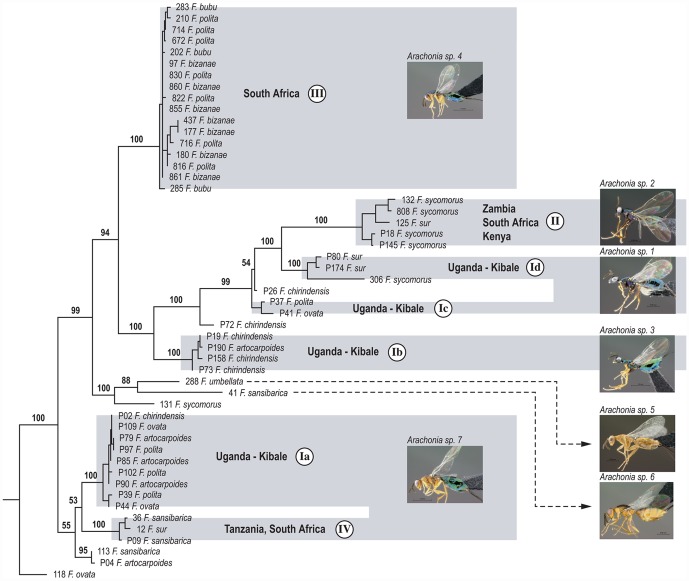
Bayesian consensus phylogram of *Arachonia* inferred using *COI*, *Cytb*, and *EF-1α* gene fragments. Taxon codes and *Ficus* species associations are shown for terminal branches. Grey boxes connect morpho-species with pictures of *Arachonia sp*. 1 to 7. Circled Roman numerals are references to haplotype groups (Ia to IV). Posterior probabilities are shown for backbone divergences only. Posterior probability support for nodes >90% are given in Figure S3.

Our Bayesian phylogenetic inference of *Arachonia* haplotypes (Figure 3) indicates well-supported (>90) stem clades (Figure S2) that are largely consistent with the haplotype groups estimated using statistical parsimony (there are very few departures from this pattern: P17/Id; P15/II; P28/Ib; P27/Ib; P24/II). One instance invalidates the cross-section host use status of haplotype group Ic. We believe that the Bayesian inference is correct and that insufficient fragment coverage (only *Cytb*) for this specimen resulted in spurious parsimony networks in this instance. The two major *Arachonia* clades that we collected from either section *Sycomorus* or *Galoglychia*, group as sister-clades with only two instances of host-use paraphyly. The two instances of host-use paraphyly (specimens 12 on *F. sur* and 131 on *F. sycomorus*) are well supported in the phylogeny inferred using the *COI*, *Cytb*, and *EF-1α* data set (see below). The specimen 131 is a relatively divergent singleton likely to represent a single species (Figure 4). Variation of haplotype divergences within each clade (I–IV) is evident. Branch length differences within clades were mostly negligible in clade Ia and III with some divergent clades within each. Greater branch length variations within the other clades were a result of divergences between individuals collected from outside Kibale in other countries. The remaining substantial divergences between individuals are an indication of a fraction of the genetic diversity that was presumably strongly influenced by sampling bias i.e. not being able to capture all the variation present in Kibale Forest. Minimal branch lengths inferred using bifurcating trees of intraspecific relationships violate assumptions that a proportion of haplotypes can be identical. Therefore, clusters of small branches indicate population-level relationships and for all clades these include associations from between two and four *Ficus* species.

To more stringently assess putative species relationships, a subset of the sequence data including a nuclear gene fragment of *EF-1α* was used to infer a phylogeny (Figure 4). Branch length differences between major clades in the phylogenetic inference indicate that at least seven putative (genetic) *Arachonia* species are present in Kibale Forest inclusive of lineages with more intermediate divergence levels. However, genetic divergences were apparently large within some of these lineages that represent morpho-species (see below). Negligible branch length differences are evident in clades with haplotypes from groups Ia, Ib, Id, III, and IV. By contrast, posterior probability support for stem clades, sometimes consisting of one or more derived clades, was above 90 in most instances (Figure S3). The longest braches are indicative of species-level divergences although there are relatively deep genetic divergences within some morpho-species lineages. Deep divergences of this type appear to represent isolation by distance effects. Phylogenetic uncertainty also appears to influence the interpretation of some deeper divergences within morpho-species lineages. Overall, the phylogenetic inferences are consistent with statistical parsimony structuring of intraspecific levels of divergence at haplotype and nuclear genes. The largest divergences most likely represent putative species and seven of these have associations with more than one *Ficus* species.

Seven *Arachonia* species were distinguishable based on morphological assessment of the specimens included in these analyses (Table S3; Figures S4 & S5). The *Arachonia* species are distinguishable using a range of morphological characters including the relative length of the ovipositor valves; propodeal shape; density of the multiporous plate sensilla on the antennal flagellar segments; shape of the antennal anelli; position of the antennal toruli on the face; relative lengths of the forewing venation (marginal, postmarginal and stigmal veins) and colour which varies from dark bluish-black, through green, yellowish-green to brownish-yellow or plain yellow. Five of the morphological species correspond with the haplotypes (Table S3; Figure 3). The remaining two species were not present in the Kibale ecosystem and were represented by a single or two specimens. They clustered together as a sister clade to haplotype group III (Figure 3). *Arachonia* species 1 correlated with both groups Id and Ic. Similarly *Arachonia* species 7 correlated with groups Ia and IV suggesting that these two species of *Arachonia* may each represent two or more cryptic species. Together, these findings demonstrate the presence of three good morphological *Arachonia* species present in Kibale Forest during the sampling period in 2008. One of the morphological species collected in Kibale shows a deep genetic divergence between clades (haplotype groups Ic and Id) that were able to reproduce in either host species of section *Sycomorus* or *Galoglychia* and indicative of cryptic species. Morpho-species 7 (group IV), able to reproduce on two host sections, represented the broadest host range for a genetically and morphologically highly similar type.

## Discussion

This study demonstrated that *Arachonia* species in Africa have conservative host plant associations among *Ficus* subsections *Sycomorus* (section *Sycomorus*) and *Caulocarpe* (section *Galoglychia*). A population (haplotype group 1a, *Arachonia* sp. 7) present within Kibale Forest was able to reproduce in at least four *Ficus* species, and in one instance, eleven identical haplotypes were collected from these four species. A divergent population of *Arachonia* species 7 was also associated with a further two species of fig elsewhere in Southern Africa (haplotype group IV). A large majority of haplotypes were associated with a single subsection and use of more than one by a single population was rare. These relationships indicate a high degree of historical host conservatism within *Ficus* subsections with infrequent horizontal transfer between host-plant subsections and sections.

Morpho-species 7 (haplotype groups Ia & IV) was collected from five *Ficus* species within section *Galoglychia* and one species from section *Sycomorus*. A relatively deep genetic divergence within this distinct morpho-species (Figure 4) suggests restricted host-use and divergence between species characteristic of habitat in Uganda, Tanzania or South Africa. The deep divergence within morpho-species 1 that is split between populations on *Sycomorus* or *Galoglychia* was estimated from the collection made in Kibale. The split therefore indicates host-subsection conservatism, as does the split between these subsections in the haplotype tree in Figure 3 made from collections over Southern Africa. An *Arachonia* population (Ia) was collected from four *Ficus* species in Kibale Forest. This pattern strongly supports incomplete host-switching and a multiple species host-range. We uncovered five *Arachonia* species distributed through Southern Africa that were able to reproduce on more than a single species (Figure 4). We expect sampling bias in this study underestimated the true extent of the host-plant species range of a given population, but still show compelling evidence of host-plant conservatism within subsections. We were unable to capture all possible wasp species associated with a particular *Ficus* species because not all receptive individual trees in a patch can be located by all wasp species that specialise on it, and not all syconia were collected. Our findings demonstrate host plant species-specificity of *Arachonia* in respect to pollinating species, is consistent with the more relaxed host conservatism displayed by parasitoids in general.

This study shows that selection for parasitoid fig wasp host-plant species conservatism is concomitant with *Ficus* subsection and that parasitoids have broader host-ranges than pollinator and possibly non-pollinator galling species. However, this does not imply that all host wasp species are present, or attacked, at the patch scale. Nor must parasitoids have compatible reproductive phenology with all host wasp species that specialize across the subset of their *Ficus* hosts. The net or effective host wasp range during a given reproductive cycle at a patch need only be the sum of the proportion of host wasp species available from any ‘compatible’ *Ficus* species in the patch. Host *Ficus* range and apparent ‘flexibility’ in host wasp species implies that the phenotype's parasitoids interact with in the course of locating a fig and ovipositioning, are quantitatively and qualitatively different than host wasp species. These differences are partly dependent on the form of parasitism, cues for locating hosts, and external and internal oviposition strategies that present different phenotpye interactions. Additionally, immune responses to parasitism common in Hymenoptera [42] might further limit host-plant ranges to host wasp defence characteristics associated with the parasitoid host-plant range. Taken together, parasitism of the pollination mutualism is an evolutionarily stable strategy characterized by different forms of specialization that appear to intersect predominantly at phenotypes of *Ficus*.

Fig trees rely exclusively on pollination by a wasp that is technically a seed parasite. Yet, a balance is maintained between pollinator and plant fitness [12]. Janzen [9] proposed that parasitoids limit the number of pollinators able to carry pollen to another fig. One mechanism that should permit host-parasitoid stability is the proposition that attack rates by parasitoids are mitigated by selection on pollinating hosts to use inner ovules out of the reach of parasitoid ovipositors [36]. Pollinator galls are mostly apparent at the innermost layer of ovules that are supported by the longest pedicels and have shorter styles. Variation in style length is believed to be a result of trade-offs between pollinator fecundity and fig seed set and selection on internally ovipositioning wasps to avoid outer ovules and greater risk of parasitoid attack [43]. There should also be selection for low rates of oviposition by parasitoids since exiting the fig is dependent on agaonid males making holes in the syconium wall. Janzen suspected fig species-specificity by parasitoids should be selected for in order to synchronize developmental times of the internal gallers such that holes are available for escape. Our findings suggest that synchronization of parasitoid and host reproductive phenology is characterized by asymmetrical species-specificity. Additionally, the stability of coexisting populations of parasitoid and pollinating fig wasps is facilitated by differential rates of parasitism between parasitoid genera on host wasps [44]. Competition between pollinator foundresses for oviposition space that reduces the production of galls [44] might also be intensified when there is selection for inner ovule use. However, realized species-specificity, the intensity of attack rates, and oviposition competition will vary across space and time if interactions are a function host patch heterogeneity and resource undersaturation [18].

The relationship between host densities and patch heterogeneity has been suggested to facilitate stability in host-parasitoid populations [45]. The ability to use multiple host species does not necessarily predict parasitoid presence on all available *Ficus* species within a patch containing host species. Our findings indicate that parasitoids do not recruit to all available figs within a patch (Figure 2). This suggests other factors such as host-plant detection and resource densities influence interactions [18], [46]. Figure 2 shows that at least several *Ficus* species were receptive to oviposition at the same time in Kibale Forest; yet not all putative parasitoid species were collected from all potential host trees. Under-sampling each individual tree could account for such a pattern. However, we show that during August 2008 very few individuals of each *Ficus* species were receptive to or were releasing wasps in this period. For example, species such as *F. chirindensis* had only 1 of 22 individuals with figs, and 2 of 28 *F. artocarpoides*, and 1 of 7 *F. sur* individuals within Kibale were releasing fig wasps over the sampling period. It is a realistic presumption that fig wasp cycling within Kibale could not occur without immigration from outside the forest. There are many variables that affect which fig wasp species recruit to a particular fig crop. The asynchrony of syconia development combined with variation in population densities limit recruitment to receptive figs. The wasp species assemblage that is available to a receptive fig crop very much depends on regional scale influences on local scale processes [47], [48], [49]. A strong correlation between regional and local fig wasp species diversity has been previously demonstrated [18]. Volatile organic compound production by host plants that are specific to particular herbivores have been shown to be exploited by parasitoids to locate their prey [50]. Parasitoids can be responsive to the same cues used by their prey species for locating and identifying hosts [51], [52]. Likewise, volatile organic compounds have been shown to be responsible for maintaining pollinator-host specificity and likely used as a cue by non-pollinating fig wasps [29], [30], [31]. However, pollinator and parasitoid wasps might respond differently to volatile cues in limiting recruitment [33]. More elaborate antenna morphology of pollinators compared to parasitoids suggests increased sensitivity in volatile odor detection [33]. Parasitoids might not be as finely tuned to specific odors, but instead use a combination of cues. In addition, the aerial pool of fig wasp species should vary tremendously over time and space. Therefore, stochastic processes partly determine whether a particular fig crop will recruit all or only part of the potential wasp assemblage that could be associated with that host tree. Predation or parasitism on sycoryctines by a diversity of organisms has been observed [36]. Ant presence certainly reduces the parasitism rate [53] though it is unlikely that predation on parasitoids would completely exclude a species from a given crop. Somewhere a fig wasp is going to slip through and successfully oviposit even if all the figs are crawling with ants as we observed on *F. artocarpoides* in Kibale Forest. Competition for ovules/galls among fig wasps might play a role in local abundance patterns. Grover & Holt [54] theorized that two competing prey species should coexist if one participant is more strongly resource-limited and the other is more strongly limited by a predator. Pollinator competition for oviposition sites might be alleviated via parasitoid predation of other non-pollinating wasps. For example, the presence of prey species has been shown to change the abundance of another natural enemy that increased competition on a second prey species [55].

Processes explaining spatiotemporal variation in population occurrence are not well developed in the empirical literature [56]. Modelled scenarios between partners in a mutualism and antagonistic associations [2] have emphasized fitness trade-offs relating to spatial fluctuations in population size, dispersal characteristics, and host visitation frequency. Resource densities encountered by parasitoid fig wasps are presumably far less compared to the distribution of *Ficus* targeted by their galler hosts. Non-pollinating species are able to feed as adults, have longer life spans outside the syconium, and larger body sizes. These traits enhance dispersal ability relative to the pollinators [38]. Additionally, host-plant visitation by all fig wasps should be limited by resource patch heterogeneity. Source-sink theory [57] and top-down versus bottom-up [58] hypotheses have been inadequate in explaining some observations. Habitat preference and quality can be independent and is further complicated by spatial variation in primary and secondary hosts. Parasitoids are able to reproduce in *Ficus* that likely have interspecific variation in volatile signatures [33] so parasitoids might be interpreting different qualities of host-plant volatile cues that are comparatively similar because they are produced by related species, or those associated with a particular habitat. Selection for host ranges in parasitoids might therefore be constrained by a wider range of traits associated with several host-plant species or even at the habitat level [59], compared to the species they attack. Our results show that single *Arachonia* species are distributed widely over Southern Africa and associated ecosystems. *Ficus* have adapted to hydric and xeric ecosystems, and this relationship shows phylogenetic correspondence with habitat type [60]. This suggests *Ficus* patch connectivity between habitat types such as forest, savanna, and desert, limits gene flow among fig wasps. Each habitat likely possesses *Ficus* with similar phenotypes among species suited to local conditions. For instance, the size of syconium or hardness of the fig wall differs between *Ficus* in contrasting habitats that are partially dependent on abiotic factors such as climate and water availability [61], [62].

Our findings indicate that parasitoids attack host wasp species that specialize on one or more of several *Ficus* host species and improves our interpretation of the ecology and evolution of the *Ficus*-pollinator mutualism. We now have a more complete perspective on the distribution of parasitoid populations in relation to host *Ficus* species and the wasp species they attack. This form of specialization characterizes ecological interactions that have persisted over evolutionary periods. Both incomplete host-switching within the subsection level and host preference switching at least between subsections contribute to parasitoid fig wasp diversification. Host-plant conservatism by parasitoids suggests host-plant traits, and possibly direct and indirect interactions with host wasp lineages, constrain parasitoid evolution. It is highly likely that *proto-fig-wasps* were able to utilise ancestors of *Ficus* in a manner that differs from contemporary biological organization. The sequence of independent colonisations of *Ficus* ancestors by different chalcid lineages suggests that the mutualism is robust to changes in community organization in respect to interactions with antagonistic phenotypes. Alternatively, the mutualism itself might be a result of these influences, existing as another form of commensalism previously. External oviposition by parasitoids targeting fig wasps that oviposit internally and produce galls should be a derived characteristic of the mutualism. Reconstruction of external and internal oviposition character evolution by different fig wasps and other closely related chalcid lineages should generate new hypotheses explaining the evolution of functional organisation of fig wasp communities and mutualisms.

## Materials and Methods

### Taxon sampling

We included the parasitoid fig wasp genus *Arachonia* as the ingroup for this study. The parasitoid fig wasp sister-genus *Sycoryctes* Mayr was used as the outgroup, as this genus is recognised as the sister-clade of *Arachonia* Joseph. *Arachonia* species are known from *Ficus drupacea* Thunberg (section *Malvanthera*) in India; *Ficus stupenda* Miquel (section *Conosycea*) in Borneo [63]; *Ficus benghalensis* Linnaeus (section *Urostigma*) in India [64]; and *Ficus annulata* (section *Urostigma*) in Malaysia [65]. It was subsequently established that the *Sycoryctes* species reported by Compton in [36] was an *Arachonia* species [28]. The *Arachonia* have a propodeum (the last dorsal segment of the mesosoma "thorax" before the metasoma "abdomen") that is as long as wide and shaped more like a bowl whereas the *Sycorcytes* have a transverse propodeum that is thin and narrow (much shorter than wide). See key to Sycoryctinae genera: http://www.figweb.org/Fig_wasps/Pteromalidae/Sycoryctinae/Key/index.htm. In total, 145 separate *Arachonia* individuals from seven *Ficus were sequenced*. Eighty-four of these individuals *were* from Kibale Forest. The specimens were initially designated using *Ficus* species affiliation and cuticle colour. Voucher specimens were deposited at Iziko South African Museum collection (Cape Town). To maximise DNA sequence variation capture and to compare to known phylogenetic species, *Arachonia* from previous collections [27], [28] were also sequenced. All additional taxa were from collections in Nigeria, Uganda, Kenya, Zambia, Tanzania, and South Africa, collected between 2005 and 2011, from nine *Ficus* species.

Fig and wasp larval development takes between three and 20 weeks, with parasitoids arriving and targeting other fig wasp guilds towards the very end of fig development [66]. We assume that the fig wasps reared from the collections made over the month census comprised no more than two generations; foundresses and dispersers. Between approximately 100 and 40 syconia per individual tree were used to rear wasps from. Syconia were removed from the tree and placed into a sterile plastic sealable bag. Care was taken to select syconia that were at different stages of development in order to capture as many fig wasp species associated with a particular *Ficus* species. Within 8 hours of being removed from the tree, the syconia collections were placed into rearing canisters. Up to 10 syconia were placed into a sterile cardboard tube with a replaceable and transparent collection jar at one end and sealed. As wasps emerge from the syconia, they migrate toward the jar in to the direction of the light. Wasp fauna were removed from the jar and placed live into >95% ethanol approximately every 8 hours. Rearing canisters produced wasp collections from between 1 and 5 days. Each syconium can potentially contain hundreds of galled ovules. This varies between syconia of different size and wasp presence. Tropical fig species typically have very large syconia. Our collections produced thousands of fig wasps that were sorted using a dissecting microscope at the Museum subsequent to the fieldwork.

We collected fig wasps from all species of *Ficus* that produced fig crops in the Kibale Forest National Park in Uganda (0° 30′ 0″ N, 30° 24′ 0″ E) over a one-month period in August 2008 before the beginning of the wet-season true. In this regard, our collection represents a snapshot of the fig wasp ecology of Kibale Forest. In order to maximise the rate of encountering the same parasitoid species on several hosts at a local ecological scale, exhaustive sampling over a one month period of all fig trees in a large forest patch that were releasing wasps was conducted. Access to nearly all parts of the forest was possible due to a grid system of tracks created to census primate populations. We located 116 individual *Ficus* trees (20 species, 4 of these unknown) in Kibale Forest National Park situated in Uganda. Of these, we reared wasps from 11 individual trees comprising 9 species that were releasing wasps during the sampling period in August (Table 1). Only 6 of these 9 species released *Arachonia* (Table S1). *Ficus asperifolia* was present in substantial numbers in the secondary forest components of Kibale (estimated >50 individuals). This dioecious species was abundant and occurred as a small hemi-epiphyte clearly associated with disturbed vegetation. We did not keep accurate locality records of *F. asperifolia* because it was difficult to count often being found in close proximity to one another not easily discerned as individuals. *Arachonia* have not been reared from and are unlikely to use this divergent species as a host that typically produce extremely low numbers of divergent wasp fauna. However, *we reared* a wasp collection from *F. asperifolia* and confirmed this observation.

To address ecological associations between fig wasp taxa, we produced an inventory of fig wasps associated with the *Ficus* species we collected (Table S2). This inventory is a summary of species that have been reared from *Ficus* species over all our collections and not necessarily from Kibale in 2008. Kibale Forest includes old growth and adjacent secondary forest covering an area of approximately 500 square kilometres in size. The forest is situated close to the eastern-most edge of the Congo Basin and includes endemic West African flora. Kibale is surrounded by expanses of habitat used for traditional agriculture (www.uwa.or.ug/kibale.html) that supports lower densities of *Ficus*. In order to provide maximal representation of genetic variation, the *Arachonia* sequence data from the forest patch was augmented with all other sources of available data from specimens collected between 2005 and 2011 from Southern Africa (Table S1). All necessary permits were obtained for the collection of specimens in nature reserves and national parks (Uganda Wildlife Authority File No. NS 164 and a No. 138/07/1; Uganda Wildlife Authority File No. NS 214 and a No. 357/07/1; Isimangaliso Wetland Park Authority Permit number 5017/2007).

### DNA sequencing

We sequenced fragments of mitochondrial DNA (mtDNA) *cytochrome oxidase I* (*COI*), *cytochrome b* (*Cytb*) and nuclear DNA (nDNA) *elongation factor one alpha* F2 copy (*EF-1α*) gene regions in one direction only. The head and thorax of single whole fig wasps, preserved in >96% ethanol, were used for DNA extractions and sequencing. A QIAGEN® DNEasy Kit was used for all DNA extractions following the manufacturer's protocol. SuperTherm Taq DNA Polymerase 250U was used in the PCR reactions. Amplifications of mitochondrial DNA were undertaken using the following: 94°C, 30 seconds denaturation; 50°C (mtDNA) or 56°C (nDNA), 1.5 minute annealing; 72°C, 1.5 minute extension for 45 cycles; with a final cycle of 72°C, 7 minute extension. The polymerase enzyme required a 94°C, 3 minutes incubation period for the first cycle only. The PCR mixture was a 25 µl reaction including: 0.2 µl of 5 U/µl of polymerase, 2.5 µl (10 mg/ml) of dNTPs0, 1.0 µl (0.2 pmol/µl) of each primer, an unknown concentration of template DNA, and 2.5 µl 10X buffer and 13.3 µl of distilled water, or 2.0 µl 10X buffer and 13.8 µl of distilled water respectively. Approximately 630 base pairs of the gene for *COI* was amplified using primer pair sets: COI-070368 forward primer 5′ TTA TCT TTA CCA GTA TTA GC 3′ with COI-070029 reverse primer 5′ AAT GTT GAG GGA AAA ATG T(CT) 3′ [27]. Approximately 400 base pairs of the *Cytb* gene fragment were amplified using Cytb-070330 forward primer 5′ CTA CCA TGA GGA CAA ATA TC 3′ with Cytb-070326 reverse primer 5′ (AG)GA AT(TA) GAT CG(TA) A(AG)A AT(TA) GC 3′ [27]. Up to 500 bases of the *EF-1α* gene fragment was amplified using EF1a-080588 forward primer 5′-GGT CTT GGA CAA ACT GAA GG-3′ (McLeish unpublished) with EF1a-073534 reverse primer 5′-TTG TC(AG) GT(TG) GG(CT) CTG CT(TG) GG-3′. Sequences were aligned by eye against pre-existing sequence data of other sycoryctine fig wasps. Positions with ambiguous sites were coded with IUPAC symbols. Sequences were submitted to GenBank under the accession numbers JQ838891 to JQ838998 and JQ839017 to JQ839124 (Table S1).

### Statistical parsimony analysis and AMOVA

As bifurcating trees can violate assumptions of intraspecific genetic relationships because some individuals are identical, network approaches account for both intra- and interspecific processes. We inferred haplotype networks using *COI* and *Cytb* mtDNA sequence data (together 1032 bp's) to estimate genealogical associations in relation to *Ficus* species ranges. Ranked uncorrected-p and *K2P* distributions of *COI* genetic distances were estimated to provide a barcoding reference frame of divergences represented in the study (Figure S6). We used the structure of parasitoid haplotype networks in relation to the *Ficus* species they were reared to assess the level of species specificity. Random observations of host-use were expected to reflect more erratic *Ficus* preference and ability to switch species more readily. Alternatively, more restricted specificity is expected to produce parasitoid haplotype structuring that corresponds to a narrow range of species. Most of the wasp collections were sampled during a one-month period in the same locality. Therefore, low-level genetic divergences at these loci were expected as well as species level divergences from specimens collected outside Uganda and in different years. We used statistical parsimony to partition the *COI* and *Cytb* sequence data into independent haplotype networks that are connected by non-homoplasious mutations. The maximum number of single substitutions among haplotypes (the connection limit) preceeds the connection of haplotypes into a network differing by increasing numbers of single site changes [41]. We inferred haplotype to be able to estimate putative genetic species [67] to compare with the morphological appraisal as well. We used TCS [68] and the *CO1* and *Cytb* mtDNA sequence data to generate haplotype networks with a 95% connection limit probability under statistical parsimony. Gaps were treated as missing and no connection limit step priors were set. To estimate genetic differentiation amongst haplotype groups, *F_ST_* and *P*-values (0.05 significance level) were estimated using analyses of molecular variance (AMOVA) implemented in Arlequin version 3.0 [69]. The *F_ST_* coefficient is the proportion of the genetic variance within a subpopulation (*S*) relative to the total genetic variance (*T*). A high *F_ST_* (closer to 1 than 0) implies substantial differentiation among groups and was expected under the hypothesis of populations representing putative species. The *P*-value of each test is the proportion of permutations resulting in an *F_ST_* value larger or equal to the observed proportions. We estimated a gamma distribution prior of 0.5 using Modeltest version 3.0 [70] and 10,000 permutations to estimate *F_ST_* and *P-*values.

### Putative species delimitation and taxonomy

The Generalised Mixed Yule Coalescent (GMYC) approach [71] uses a maximum likelihood approach to identify genetic clusters representing independently evolving entities. This is done using a likelihood test of a mixed model that estimates the shift from speciation to within-population branching of an ultrametric tree according to Yule pure-birth [72] and neutral coalescent [73] models respectively. The GMYC test was implemented using the ‘R’ [74] package SPLITS (available from: http://R-Forge.R-project.org). An ultrametric tree reconstruction was generated using a strict molecular clock with gamma distributed invariant sites, GTR substitution prior, empirically estimated base pair frequencies, and unlinked codon positions implemented in BEAST v.1.4.8 [75]. Generalised time reversible (GTR), empirical base frequency, gamma plus invariant sites were selected as substitution rate model priors. An arbitrary value (10) was chosen as a convenient scale to calibrate the ingroup common ancestor node of the tree that was converted to a relative time scale for interpretation. The outgroup was pruned before R analyses. The Markov chain was run for 20 million generations, sampling each chain every 1000 trees. A burnin of the first 7500 trees in the Markov chain was conducted in TreeAnnotator version 1.4.8 [75].

As haplotypes are grouped according to similarities, bifurcating trees cannot always represent intraspecific relationships. However, the inherent low divergences associated between conspecifics will cause them to cluster and is useful for detecting like types. Poor statistical support (hard polytomies) at nodes within population clusters is expected. Phylogenetic inference is useful in assessing “exclusivity” of populations as monophyletic clades [76]. A two-step procedure was used to more stringently assess deeper species-level divergences inferred from the haplotype tree. First, a phylogeny of all 145 *Arachonia* specimens was inferred using the mtDNA sequence data to identify (exclusivity) putative population-level clades. A second inference was conducted to infer a phylogeny using a subset of 51 *Arachonia* specimens with the inclusion of the *EF-1α* nuclear DNA marker. The second analysis comprised taxa having unique *Ficus* species associations and without multiple exemplars of the same *Ficus* association of the same inferred population. A Bayesian approach was implemented using MrBayes 3.1.1 [77] and was used to infer the phylogenies. The sequence data was partitioned into 1^st^, 2^nd^, and 3^rd^ codon positions with both mtDNA and mtDNA-nDNA datasets. Substitution model priors are explained in [27]. Four Markov chains were run for 40 million generations, sampling each chain every 1000 trees. A consensus phylogram as well as a consensus tree indicating posterior probability node support values was generated from post-burnin of 35000 generations. Convergence was assessed using the MCMC Tracer Analysis Tool v.1.4.1 [78] by plotting the log likelihoods to assess the point in the chain where stable values were reached and with the standard deviation of split frequencies of all runs.

Species delimitation using the molecular approaches was assessed with appraisal of morphological variation among and within putative species. Specimens were dried from ethanol, point mounted, and examined using a Wild stereo microscope. Images were produced using the EntoVision multi-stacking imaging system. This system included a Leica M16 zoom lens attached to a JVC KY-75U 3-CCD digital video camera that fed image data to a notebook computer. The program Cartograph 5.6.0 was then used to merge an image series into a single in focus image. Lighting was achieved using techniques summarized in [79], [80], [81].

## Supporting Information

Figure S1 **Ultrametric phylogeny of** ***Arachonia*** **haplotypes inferred using a strict molecular clock implemented in BEAST.** Red clades fall within the neutral coalescent model for intraspecific branching. The shift from branching under the Yule pure birth model was estimated using a mixed model likelihood test (*P*<0.001) called the generalized mixed Yule coalescent (GMYC) implemented using SPLITS.
(TIF)

Figure S2 **Bayesian consensus haplotype phylogeny of** ***Arachonia*** **.** The phylogeny was inferred using *COI*, and *Cytb* gene fragments and shows posterior probabilities above 90%.
(TIF)

Figure S3 **Bayesian consensus phylogeny of the genus** ***Arachonia*** **.** The phylogeny was inferred using *COI*, *Cytb*, and *EF-1α* gene fragments and showing posterior probabilities above 90%. Terminal taxa are shown as the isolate code followed by the *Ficus* species the specimen was collected from.
(TIF)

Figure S4 ***Arachonia*** **species, lateral habitus.** A: species 1; B: species 2; C: species 3; D: species 4.
(TIF)

Figure S5 ***Arachonia*** **species, lateral habitus.** A: species 5; B: species 6; C: species 7; D: species 4 (repeated for direct comparison with the similar species 7).
(TIF)

Figure S6 **Ranked pair-wise uncorrected p and K2P COI distances for all specimens sequenced.** Dashed lined indicate either the first instance of an interspecific pair-wise association or an intergeneric association. Note that both cases occur together in the distribution after the second dashed line.
(TIF)

Table S1 **Arachonia specimens, voucher codes, location and DNA sequence assession numbers.**
(DOC)

Table S2 **An inventory of potential host fig wasp species specialising on the** ***Ficus*** **species from which** ***Arachonia*** **were collected in this study.** The literature suggests that Sycoryctinae target pollinators mostly and other non-pollinator species only infrequently [82], [83]. There is no hard evidence supporting this and we suspect that sycoryctines equally target the sycoecines and otitesellines associated with section *Galoglychia*. *Arachonia* species might also be attacking the Sycophaginae belonging to section *Sycomorus* in addition to the pollinators. The ratio of parasitoid fig wasp genera to other pteromalids and the agaonids is potentially 2∶1 for each *Ficus* species from which collections were made in Kibale. However, relatively smaller parasitoid population sizes and differences in species diversity might instead be a clue to fundamentally different evolutionary diversification processes [84]. Typically, phytophagous insect species are more abundant than parasitoid species [85], [86] and are also attacked by more than one parasitoid species [87]. These observations are consistent with our records and other fig wasp studies. Compton and colleagues [88] showed that the ratio of pollinator to non-pollinator fig wasp abundance in forest patches in Asia was approximately 3∶1. The Epichrysomallinae comprised between 45% and 75% of all the non-pollinator sub-families with fewer still of the Otitesellinae and Sycoryctinae that were in roughly equal abundance followed by the Sycoecinae and lastly the Sycophaginae. The Epichrysomallinae are gallers of fig seeds and ovules [89] and are parasitised by the Eurytomidae. Weiblen's [83] review shows a food web summary among parasitoids, other fig wasps, and *Ficus*. The trophic interactions indicate the Agaoninae and Sychophaginae as prey species of the Sycoryctinae, but did not recognise those between the Otitesellinae and Sycoecinae. Infrequent interactions with the Otitesellinae have been observed [89].
(DOC)

Table S3 **Morphological delimitation of** ***Arachonia*** **species collected in this study showing their haplotype affinities.**
(DOC)
