# Supplementary figures and images for: Host-Plant Species Conservatism and Ecology of a Parasitoid Fig Wasp Genus (Chalcidoidea; Sycoryctinae; Arachonia)
Source: PLoS One. 2012 Sep 10;7(9):e44804. doi: 10.1371/journal.pone.0044804 (PMC3438170; doi:10.1371/journal.pone.0044804)

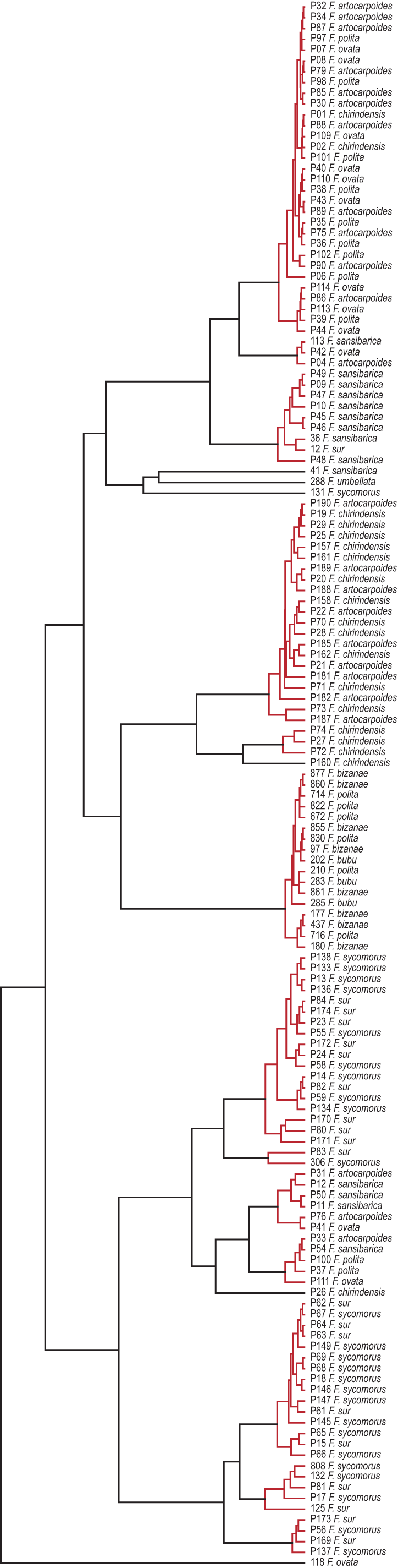

Supplement: Figure S1 — Ultrametric phylogeny of Arachonia haplotypes inferred using a strict molecular clock implemented in BEAST. Red clades fall within the neutral coalescent model for intraspecific branching. The shift from branching under the Yule pure birth model was estimated using a mixed model likelihood test (P<0.001) called the generalized mixed Yule coalescent (GMYC) implemented using SPLITS. (TIF) [file pone.0044804.s001.tif]

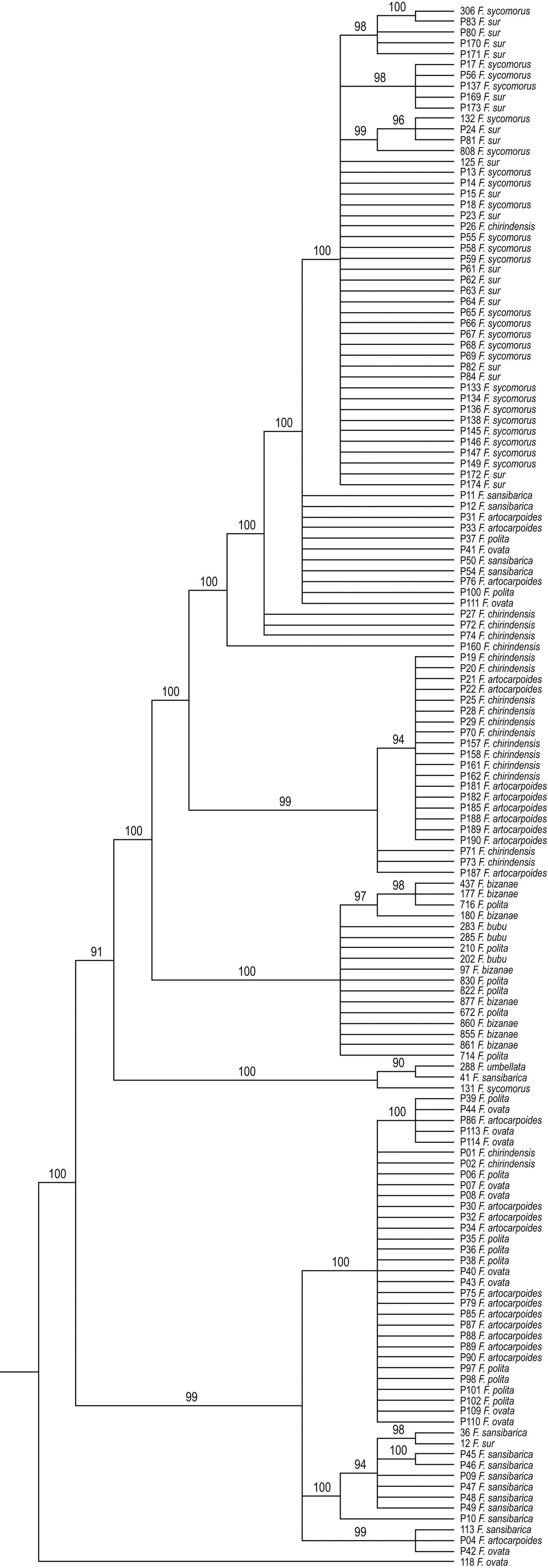

Supplement: Figure S2 — Bayesian consensus haplotype phylogeny of Arachonia . The phylogeny was inferred using COI, and Cytb gene fragments and shows posterior probabilities above 90%. (TIF) [file pone.0044804.s002.tif]

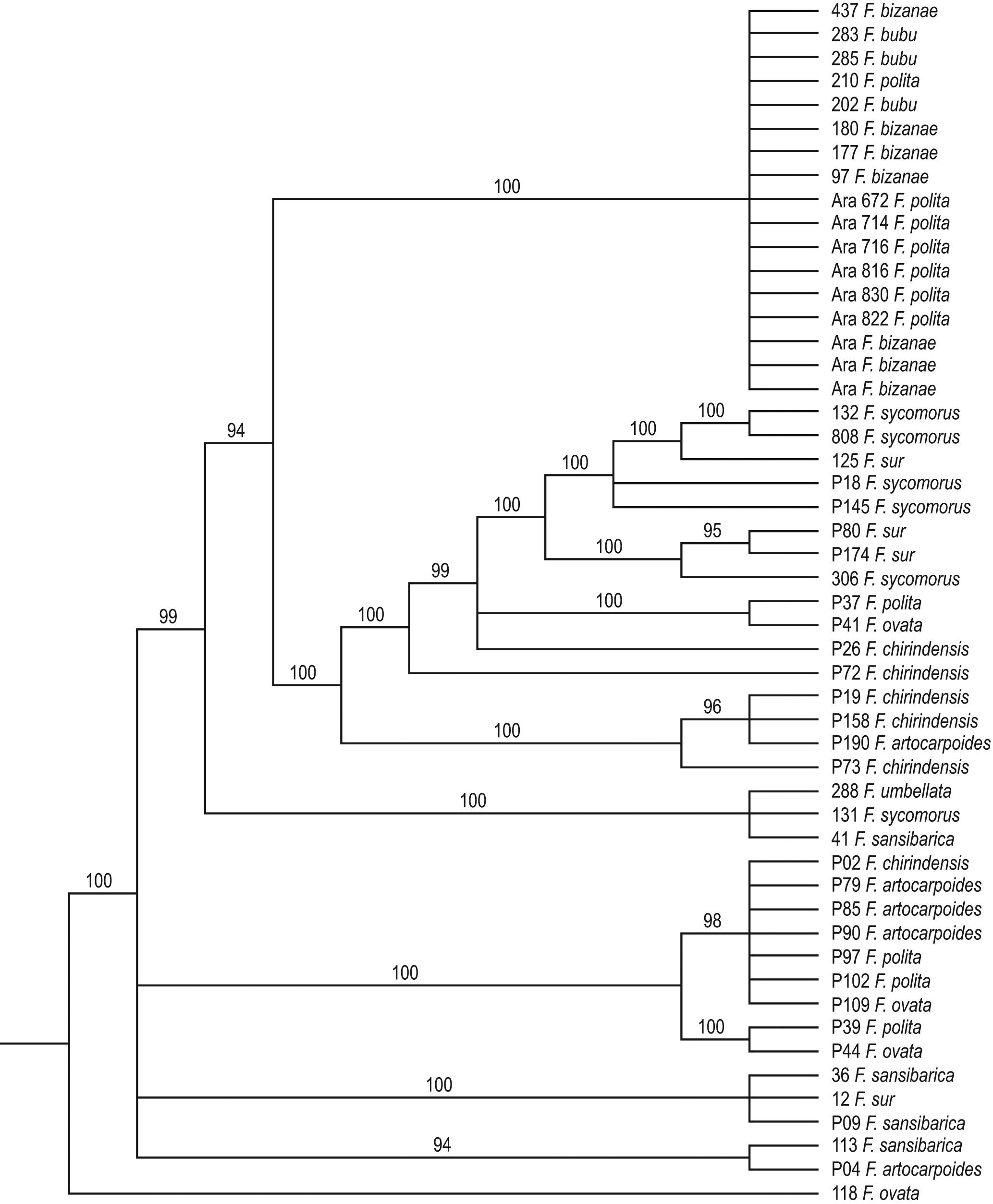

Supplement: Figure S3 — Bayesian consensus phylogeny of the genus Arachonia . The phylogeny was inferred using COI, Cytb, and EF-1α gene fragments and showing posterior probabilities above 90%. Terminal taxa are shown as the isolate code followed by the Ficus species the specimen was collected from. (TIF) [file pone.0044804.s003.tif]

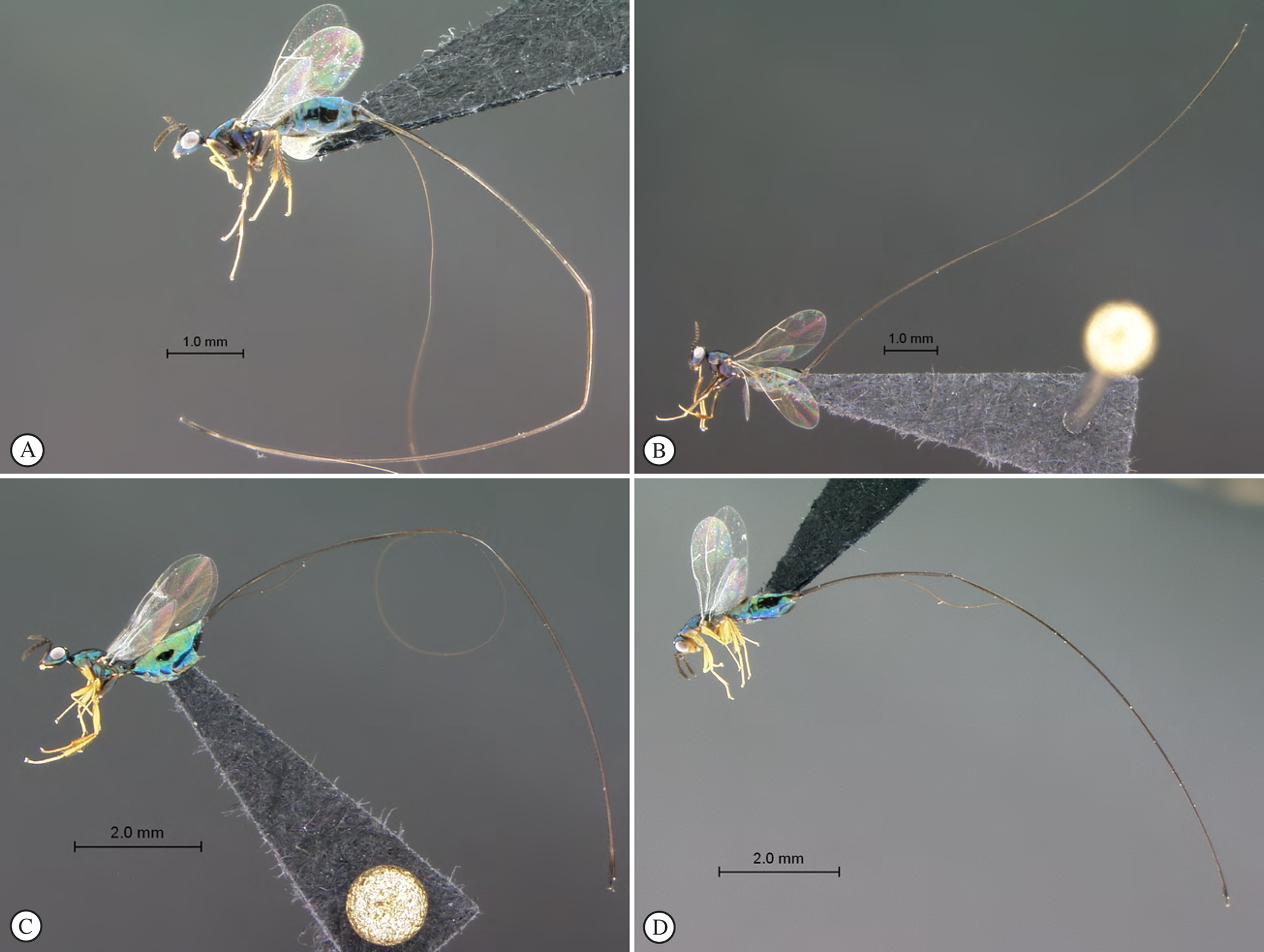

Supplement: Figure S4 — Arachonia species, lateral habitus. A: species 1; B: species 2; C: species 3; D: species 4. (TIF) [file pone.0044804.s004.tif]

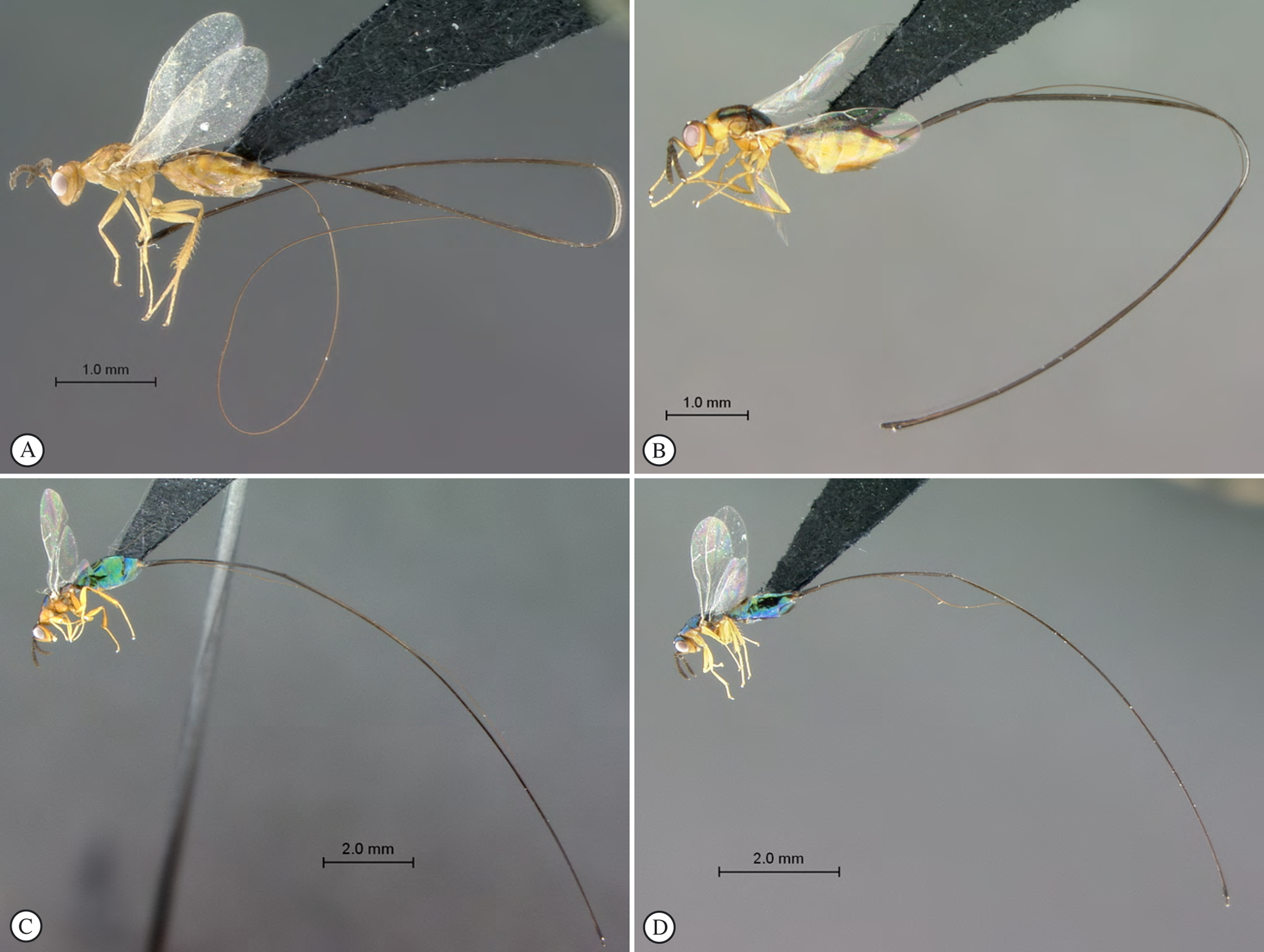

Supplement: Figure S5 — Arachonia species, lateral habitus. A: species 5; B: species 6; C: species 7; D: species 4 (repeated for direct comparison with the similar species 7). (TIF) [file pone.0044804.s005.tif]

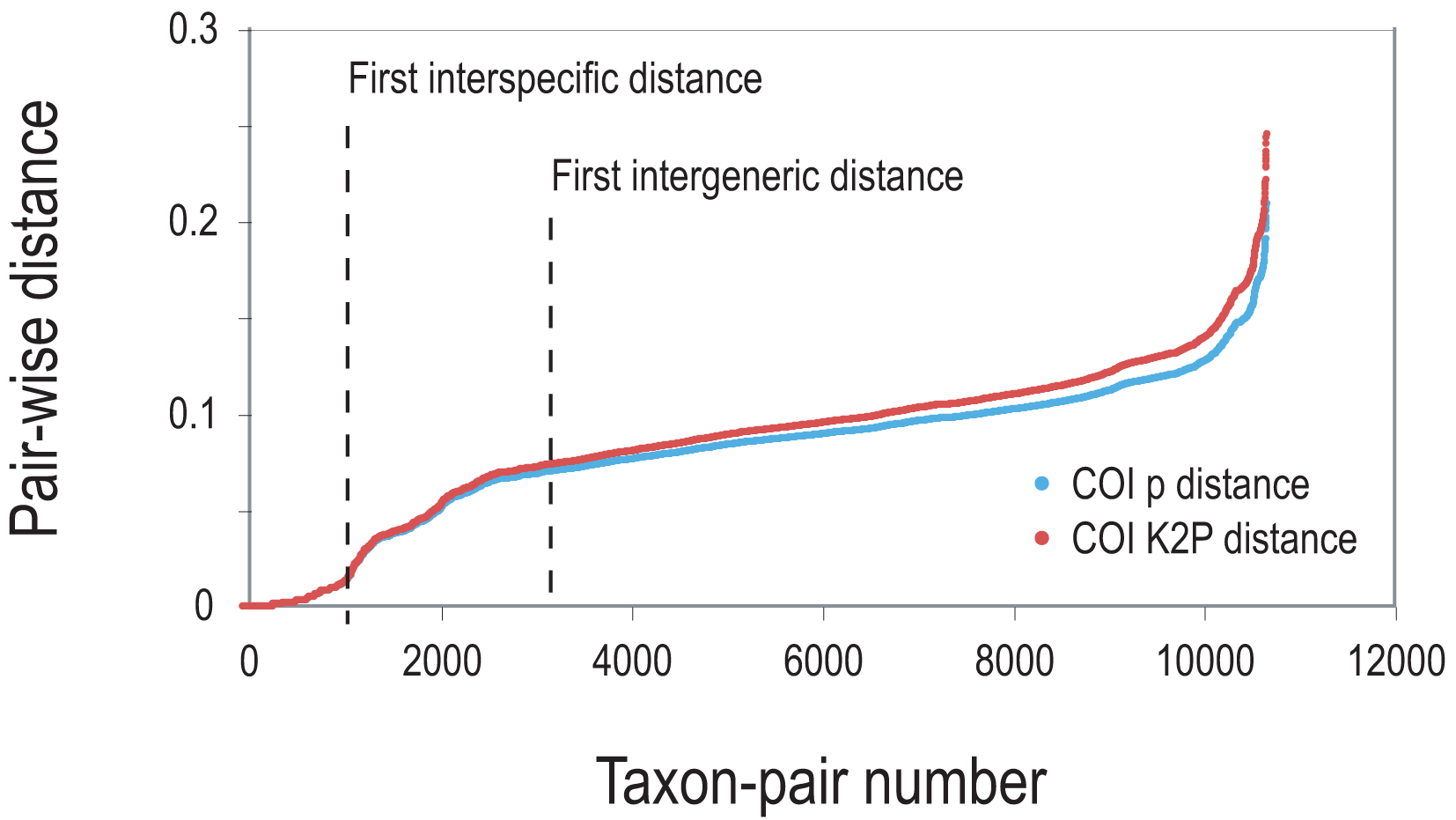

Supplement: Figure S6 — Ranked pair-wise uncorrected p and K2P COI distances for all specimens sequenced. Dashed lined indicate either the first instance of an interspecific pair-wise association or an intergeneric association. Note that both cases occur together in the distribution after the second dashed line. (TIF) [file pone.0044804.s006.tif]
