# Supplementary material for: Host-Plant Species Conservatism and Ecology of a Parasitoid Fig Wasp Genus (Chalcidoidea; Sycoryctinae; Arachonia)
Source: PLoS One. 2012 Sep 10;7(9):e44804. doi: 10.1371/journal.pone.0044804 (PMC3438170; doi:10.1371/journal.pone.0044804)
Supplement: Table S1 — Arachonia specimens, voucher codes, location and DNA sequence assession numbers. (DOC) [file pone.0044804.s007.doc]

**Table S**1: Arachonia specimens, voucher codes, location and DNA sequence assession numbers.

| **Sample code** | ***Ficus* species** | ***Ficus* subsection** | **Voucher code** | **Location** | **Date** | **Accession No. COI** | **Accession No. Cytb** | **Accession No. EF-1a** |
| --- | --- | --- | --- | --- | --- | --- | --- | --- |
| P01 | *F. chirindensis* | *Caulocarpae* | UG08-F111 | Uganda | Aug. 2008 | JQ838891 | JQ839017 | NA |
| P02 | *F. chirindensis* | *Caulocarpae* | UG08-F111 | Uganda | Aug. 2008 | JQ838892 | JQ839018 | JQ910996 |
| P04 | *F. artocarpoides* | *Caulocarpae* | UG08-F124 | Uganda | Aug. 2008 | JQ838893 | JQ839019 | JQ910997 |
| P06 | *F. polita* | *Caulocarpae* | UG08-F178 | Uganda | Aug. 2008 | JQ838894 | JQ839020 | NA |
| P07 | *F. ovata* | *Caulocarpae* | UG08-F183 | Uganda | Aug. 2008 | JQ838895 | JQ839021 | NA |
| P08 | *F. ovata* | *Caulocarpae* | UG08-F183 | Uganda | Aug. 2008 | JQ838896 | JQ839022 | NA |
| P09 | *F. sansibarica* | *Caulocarpae* | UG08-F212 | Tanzania | Aug. 2008 | JQ838897 | JQ839023 | JQ910998 |
| P10 | *F. sansibarica* | *Caulocarpae* | UG08-F212 | Tanzania | Aug. 2008 | JQ838898 | JQ839024 | NA |
| P11 | *F. sansibarica* | *Caulocarpae* | ZA06-F18 | Zambia | Aug. 2008 | JQ838899 | JQ839025 | NA |
| P13 | *F. sycomorus* | *Sycomorus* | UG07-Kam01 | Uganda | May 2007 | JQ838900 | JQ839026 | NA |
| P14 | *F. sycomorus* | *Sycomorus* | UG07-Kam01 | Uganda | May 2007 | JQ838901 | JQ839027 | NA |
| P18 | *F. sycomorus* | *Sycomorus* | UG08-F74 | Kenya | Aug. 2008 | JQ838902 | JQ839028 | JQ910999 |
| P19 | *F. chirindensis* | *Caulocarpae* | UG08-F111 | Uganda | Aug. 2008 | JQ838903 | JQ839029 | JQ911000 |
| P20 | *F. chirindensis* | *Caulocarpae* | UG08-F111 | Uganda | Aug. 2008 | JQ838904 | JQ839030 | NA |
| P21 | *F. artocarpoides* | *Caulocarpae* | UG08-F145 | Uganda | Aug. 2008 | JQ838905 | JQ839031 | NA |
| P22 | *F. artocarpoides* | *Caulocarpae* | UG08-F145 | Uganda | Aug. 2008 | JQ838906 | JQ839032 | NA |
| P23 | *F. sur* | *Sycomorus* | UG08-F154 | Uganda | Aug. 2008 | JQ838907 | JQ839033 | NA |
| P25 | *F. chirindensis* | *Caulocarpae* | UG08-F111 | Uganda | Aug. 2008 | JQ838908 | JQ839034 | NA |
| P26 | *F. chirindensis* | *Caulocarpae* | UG08-F111 | Uganda | Aug. 2008 | JQ838909 | JQ839035 | JQ911001 |
| P29 | *F. chirindensis* | *Caulocarpae* | UG08-F111 | Uganda | Aug. 2008 | JQ838910 | JQ839036 | NA |
| P30 | *F. artocarpoides* | *Caulocarpae* | UG08-F124 | Uganda | Aug. 2008 | JQ838911 | JQ839037 | NA |
| P32 | *F. artocarpoides* | *Caulocarpae* | UG08-F124 | Uganda | Aug. 2008 | JQ838912 | JQ839038 | NA |
| P34 | *F. artocarpoides* | *Caulocarpae* | UG08-F124 | Uganda | Aug. 2008 | JQ838913 | JQ839039 | NA |
| P36 | *F. polita* | *Caulocarpae* | UG08-F178 | Uganda | Aug. 2008 | JQ838914 | JQ839040 | NA |
| P37 | *F. polita* | *Caulocarpae* | UG08-F178 | Uganda | Aug. 2008 | JQ911029 | NA | JQ911002 |
| P38 | *F. polita* | *Caulocarpae* | UG08-F178 | Uganda | Aug. 2008 | JQ838915 | JQ839041 | NA |
| P39 | *F. polita* | *Caulocarpae* | UG08-F178 | Uganda | Aug. 2008 | JQ838916 | JQ839042 | JQ911003 |
| P40 | *F. ovata* | *Caulocarpae* | UG08-F183 | Uganda | Aug. 2008 | JQ838917 | JQ839043 | NA |
| P41 | *F. ovata* | *Caulocarpae* | UG08-F183 | Uganda | Aug. 2008 | JQ911030 | NA | JQ911004 |
| P43 | *F. ovata* | *Caulocarpae* | UG08-F183 | Uganda | Aug. 2008 | JQ838918 | JQ839044 | NA |
| P44 | *F. ovata* | *Caulocarpae* | UG08-F183 | Uganda | Aug. 2008 | JQ838919 | JQ839045 | JQ911005 |
| P45 | *F. sansibarica* | *Caulocarpae* | UG08-F212 | Tanzania | Aug. 2008 | JQ838920 | JQ839046 | NA |
| P46 | *F. sansibarica* | *Caulocarpae* | UG08-F212 | Tanzania | Aug. 2008 | JQ838921 | JQ839047 | NA |
| P48 | *F. sansibarica* | *Caulocarpae* | UG08-F212 | Tanzania | Aug. 2008 | JQ838922 | JQ839048 | NA |
| P49 | *F. sansibarica* | *Caulocarpae* | UG08-F212 | Tanzania | Aug. 2008 | JQ838923 | JQ839049 | NA |
| P55 | *F. sycomorus* | *Sycomorus* | UG07-Kam01 | Uganda | May 2007 | JQ838924 | JQ839050 | NA |
| P56 | *F. sycomorus* | *Sycomorus* | UG07-Kam01 | Uganda | May 2007 | JQ838925 | JQ839051 | NA |
| P58 | *F. sycomorus* | *Sycomorus* | UG07-Kam01 | Uganda | May 2007 | JQ838926 | JQ839052 | NA |
| P59 | *F. sycomorus* | *Sycomorus* | UG07-Kam01 | Uganda | May 2007 | JQ838927 | JQ839053 | NA |
| P61 | *F. sur* | *Sycomorus* | UG08-F27 | Zambia | Aug. 2008 | JQ838928 | JQ839054 | NA |
| P62 | *F. sur* | *Sycomorus* | UG08-F27 | Zambia | Aug. 2008 | JQ838929 | JQ839055 | NA |
| P63 | *F. sur* | *Sycomorus* | UG08-F27 | Zambia | Aug. 2008 | JQ838930 | JQ839056 | NA |
| P64 | *F. sur* | *Sycomorus* | UG08-F27 | Zambia | Aug. 2008 | JQ838931 | JQ839057 | NA |
| P65 | *F. sycomorus* | *Sycomorus* | UG08-F74 | Kenya | Aug. 2008 | JQ838932 | JQ839058 | NA |
| P66 | *F. sycomorus* | *Sycomorus* | UG08-F74 | Kenya | Aug. 2008 | JQ838933 | JQ839059 | NA |
| P67 | *F. sycomorus* | *Sycomorus* | UG08-F74 | Kenya | Aug. 2008 | JQ838934 | JQ839060 | NA |
| P68 | *F. sycomorus* | *Sycomorus* | UG08-F74 | Kenya | Aug. 2008 | JQ838935 | JQ839061 | NA |
| P69 | *F. sycomorus* | *Sycomorus* | UG08-F74 | Kenya | Aug. 2008 | JQ838936 | JQ839062 | NA |
| P70 | *F. chirindensis* | *Caulocarpae* | UG08-F111 | Uganda | Aug. 2008 | JQ838937 | JQ839063 | NA |
| P71 | *F. chirindensis* | *Caulocarpae* | UG08-F111 | Uganda | Aug. 2008 | JQ838938 | JQ839064 | NA |
| P72 | *F. chirindensis* | *Caulocarpae* | UG08-F111 | Uganda | Aug. 2008 | JQ838939 | JQ839065 | JQ911006 |
| P73 | *F. chirindensis* | *Caulocarpae* | UG08-F111 | Uganda | Aug. 2008 | JQ911031 | NA | JQ911007 |
| P74 | *F. chirindensis* | *Caulocarpae* | UG08-F111 | Uganda | Aug. 2008 | JQ838940 | JQ839066 | NA |
| P75 | *F. artocarpoides* | *Caulocarpae* | UG08-F145 | Uganda | Aug. 2008 | JQ838941 | JQ839067 | NA |
| P79 | *F. artocarpoides* | *Caulocarpae* | UG08-F145 | Uganda | Aug. 2008 | JQ838942 | JQ839068 | JQ911008 |
| P80 | *F. sur* | *Sycomorus* | UG08-F154 | Uganda | Aug. 2008 | JQ838943 | JQ839069 | JQ911009 |
| P81 | *F. sur* | *Sycomorus* | UG08-F154 | Uganda | Aug. 2008 | JQ838944 | JQ839070 | NA |
| P82 | *F. sur* | *Sycomorus* | UG08-F154 | Uganda | Aug. 2008 | JQ838945 | JQ839071 | NA |
| P83 | *F. sur* | *Sycomorus* | UG08-F154 | Uganda | Aug. 2008 | JQ838946 | JQ839072 | NA |
| P84 | *F. sur* | *Sycomorus* | UG08-F154 | Uganda | Aug. 2008 | JQ838947 | JQ839073 | NA |
| P85 | *F. artocarpoides* | *Caulocarpae* | UG08-F124 | Uganda | Aug. 2008 | JQ838948 | JQ839074 | JQ911010 |
| P86 | *F. artocarpoides* | *Caulocarpae* | UG08-F124 | Uganda | Aug. 2008 | JQ838949 | JQ839075 | NA |
| P87 | *F. artocarpoides* | *Caulocarpae* | UG08-F124 | Uganda | Aug. 2008 | JQ838950 | JQ839076 | NA |
| P88 | *F. artocarpoides* | *Caulocarpae* | UG08-F124 | Uganda | Aug. 2008 | JQ838951 | JQ839077 | NA |
| P89 | *F. artocarpoides* | *Caulocarpae* | UG08-F124 | Uganda | Aug. 2008 | JQ838952 | JQ839078 | NA |
| P90 | *F. artocarpoides* | *Caulocarpae* | UG08-F124 | Uganda | Aug. 2008 | JQ838953 | JQ839079 | JQ911011 |
| P97 | *F. polita* | *Caulocarpae* | UG08-F178 | Uganda | Aug. 2008 | JQ838954 | JQ839080 | JQ911012 |
| P98 | *F. polita* | *Caulocarpae* | UG08-F178 | Uganda | Aug. 2008 | JQ838955 | JQ839081 | NA |
| P100 | *F. polita* | *Caulocarpae* | UG08-F178 | Uganda | Aug. 2008 | JQ838956 | JQ839082 | NA |
| P101 | *F. polita* | *Caulocarpae* | UG08-F178 | Uganda | Aug. 2008 | JQ838957 | JQ839083 | NA |
| P102 | *F. polita* | *Caulocarpae* | UG08-F178 | Uganda | Aug. 2008 | JQ838958 | JQ839084 | JQ911013 |
| P109 | *F. ovata* | *Caulocarpae* | UG08-F183 | Uganda | Aug. 2008 | JQ838959 | JQ839085 | JQ911014 |
| P110 | *F. ovata* | *Caulocarpae* | UG08-F183 | Uganda | Aug. 2008 | JQ838960 | JQ839086 | NA |
| P111 | *F. ovata* | *Caulocarpae* | UG08-F183 | Uganda | Aug. 2008 | JQ838961 | JQ839087 | NA |
| P113 | *F. ovata* | *Caulocarpae* | UG08-F183 | Uganda | Aug. 2008 | JQ838962 | JQ839088 | NA |
| P114 | *F. ovata* | *Caulocarpae* | UG08-F183 | Uganda | Aug. 2008 | JQ838963 | JQ839089 | NA |
| P133 | *F. sycomorus* | *Sycomorus* | UG07-Kam01 | Uganda | May 2007 | JQ838964 | JQ839090 | NA |
| P134 | *F. sycomorus* | *Sycomorus* | UG07-Kam01 | Uganda | May 2007 | JQ838965 | JQ839091 | NA |
| P136 | *F. sycomorus* | *Sycomorus* | UG07-Kam01 | Uganda | May 2007 | JQ838966 | JQ839092 | NA |
| P137 | *F. sycomorus* | *Sycomorus* | UG07-Kam01 | Uganda | May 2007 | JQ838967 | JQ839093 | NA |
| P138 | *F. sycomorus* | *Sycomorus* | UG07-Kam01 | Uganda | May 2007 | JQ838968 | JQ839094 | NA |
| P145 | *F. sycomorus* | *Sycomorus* | UG08-F74 | Kenya | Aug. 2008 | JQ838969 | JQ839095 | JQ911015 |
| P146 | *F. sycomorus* | *Sycomorus* | UG08-F74 | Kenya | Aug. 2008 | JQ838970 | JQ839096 | NA |
| P147 | *F. sycomorus* | *Sycomorus* | UG08-F74 | Kenya | Aug. 2008 | JQ838971 | JQ839097 | NA |
| P149 | *F. sycomorus* | *Sycomorus* | UG08-F74 | Kenya | Aug. 2008 | JQ838972 | JQ839098 | NA |
| P157 | *F. chirindensis* | *Caulocarpae* | UG08-F111 | Uganda | Aug. 2008 | JQ838973 | JQ839099 | NA |
| P158 | *F. chirindensis* | *Caulocarpae* | UG08-F111 | Uganda | Aug. 2008 | JQ838974 | JQ839100 | JQ911016 |
| P160 | *F. chirindensis* | *Caulocarpae* | UG08-F111 | Uganda | Aug. 2008 | JQ838975 | JQ839101 | NA |
| P161 | *F. chirindensis* | *Caulocarpae* | UG08-F111 | Uganda | Aug. 2008 | JQ838976 | JQ839102 | NA |
| P162 | *F. chirindensis* | *Caulocarpae* | UG08-F111 | Uganda | Aug. 2008 | JQ838977 | JQ839103 | NA |
| P169 | *F. sur* | *Sycomorus* | UG08-F154 | Uganda | Aug. 2008 | JQ838978 | JQ839104 | NA |
| P170 | *F. sur* | *Sycomorus* | UG08-F154 | Uganda | Aug. 2008 | JQ838979 | JQ839105 | NA |
| P171 | *F. sur* | *Sycomorus* | UG08-F154 | Uganda | Aug. 2008 | JQ838980 | JQ839106 | NA |
| P172 | *F. sur* | *Sycomorus* | UG08-F154 | Uganda | Aug. 2008 | JQ838981 | JQ839107 | NA |
| P173 | *F. sur* | *Sycomorus* | UG08-F154 | Uganda | Aug. 2008 | JQ838982 | JQ839108 | NA |
| P174 | *F. sur* | *Sycomorus* | UG08-F154 | Uganda | Aug. 2008 | JQ838983 | JQ839109 | JQ911017 |
| P181 | *F. artocarpoides* | *Caulocarpae* | UG08-F145 | Uganda | Aug. 2008 | JQ838984 | JQ839110 | NA |
| P182 | *F. artocarpoides* | *Caulocarpae* | UG08-F145 | Uganda | Aug. 2008 | JQ838985 | JQ839111 | NA |
| P185 | *F. artocarpoides* | *Caulocarpae* | UG08-F145 | Uganda | Aug. 2008 | JQ838986 | JQ839112 | NA |
| P187 | *F. artocarpoides* | *Caulocarpae* | UG08-F145 | Uganda | Aug. 2008 | JQ838987 | JQ839113 | NA |
| P188 | *F. artocarpoides* | *Caulocarpae* | UG08-F145 | Uganda | Aug. 2008 | JQ838988 | JQ839114 | NA |
| P189 | *F. artocarpoides* | *Caulocarpae* | UG08-F145 | Uganda | Aug. 2008 | JQ838989 | JQ839115 | NA |
| P190 | *F. artocarpoides* | *Caulocarpae* | UG08-F145 | Uganda | Aug. 2008 | JQ838990 | JQ839116 | JQ911018 |
| 861 | *F. bizanae* | *Caulocarpae* | SA10-F374 | South Africa | Oct. 2010 | JQ911032 | NA | JQ911028 |
| 860 | *F. bizanae* | *Caulocarpae* | SA10-F374 | South Africa | Oct. 2010 | JQ838995 | JQ839121 | JQ911027 |
| 855 | *F. bizanae* | *Caulocarpae* | SA10-F374 | South Africa | Oct. 2010 | JQ838996 | JQ839122 | JQ911026 |
| 830 | *F. polita* | *Caulocarpae* | SA10-F277 | South Africa | Oct. 2010 | JQ838991 | JQ839117 | JQ911024 |
| 822 | *F. polita* | *Caulocarpae* | SA10-F277 | South Africa | Oct. 2010 | JQ838992 | JQ839118 | JQ911025 |
| 816 | *F. polita* | *Caulocarpae* | SA10-F277 | South Africa | Oct. 2010 | NA | JQ911033 | JQ911023 |
| 808 | *F. sycomorus sycomorus* | *Sycomorus* | SA10-F363 | South Africa | Oct. 2010 | JQ838993 | JQ839119 | JQ911022 |
| 716 | *F. polita* | *Caulocarpae* | SA10-F276 | South Africa | Oct. 2010 | JQ838998 | JQ839124 | JQ911021 |
| 714 | *F. polita* | *Caulocarpae* | SA10-F276 | South Africa | Oct. 2010 | JQ838997 | JQ839123 | JQ911020 |
| 672 | *F. polita* | *Caulocarpae* | SA10-F276 | South Africa | Oct. 2010 | JQ838994 | JQ839120 | JQ911019 |
| 437 | *F. bizanae* | *Caulocarpae* | SA06-F100 | South Africa | Nov. 2005 | HM007937 | HM008050 | HM008163 |
| 306 | *F. sycomorus gnapholocarpa* | *Sycomorus* | Ug07-Kam01 | Uganda | May 2007 | FJ886870 | FJ886944 | FJ887018 |
| 288 | *F. umbellata* | *Caulocarpae* | NI07-F01 | Nigeria | Apr. 2006 | FJ886871 | FJ886945 | FJ887019 |
| 285 | *F. bubu* | *Caulocarpae* | KN08-F124 | South Africa | Jan. 2008 | FJ886872 | FJ886946 | FJ887020 |
| 283 | *F. bubu* | *Caulocarpae* | KN08-F124 | South Africa | Jan. 2008 | FJ886873 | FJ886947 | FJ887021 |
| 210 | *F. polita* | *Caulocarpae* | KN08-F147 | South Africa | Jan. 2008 | FJ886874 | FJ886948 | FJ887022 |
| 202 | *F. bubu* | *Caulocarpae* | KN08-F124 | South Africa | Jan. 2008 | FJ886875 | FJ886949 | FJ887023 |
| 180 | *F. bizanae* | *Caulocarpae* | SA06-F102 | South Africa | Jun. 2006 | FJ886876 | FJ886950 | FJ887024 |
| 177 | *F. bizanae* | *Caulocarpae* | SA06-F100 | South Africa | Jun. 2006 | FJ886877 | FJ886951 | FJ887025 |
| 132 | *F. sycomorus gnaphalocarpa* | *Sycomorus* | ZA06-F42 | Zambia | Jun. 2006 | FJ886878 | FJ886952 | FJ887026 |
| 131 | *F. sycomorus gnaphalocarpa* | *Sycomorus* | ZA06-F42 | Zambia | Jun. 2006 | FJ886879 | FJ886953 | FJ887027 |
| 125 | *F. sur* | *Sycomorus* | ZA06-F29 | Zambia | Jun. 2006 | FJ886880 | FJ886954 | FJ887028 |
| 118 | *F. ovata* | *Caulocarpae* | ZA06-F19 | Zambia | Jun. 2006 | FJ886881 | FJ886955 | FJ887029 |
| 113 | *F. sansibarica macrosperma* | *Caulocarpae* | ZA06-F18 | Zambia | Jun. 2006 | FJ886882 | FJ886956 | FJ887030 |
| 97 | *F. bizanae* | *Caulocarpae* | SA05-F72 | South Africa | Nov. 2005 | FJ886884 | FJ886958 | FJ887032 |
| 41 | *F. sansibarica sansibarica* | *Caulocarpae* | SA05-F40 | South Africa | Nov. 2005 | FJ886885 | FJ886959 | FJ887033 |
| 36 | *F. sansibarica sansibarica* | *Caulocarpae* | SA05-F27 | South Africa | Nov. 2005 | FJ886886 | FJ886960 | FJ887034 |
| 12 | *F. sur* | *Sycomorus* | SA05-F25 | South Africa | Nov. 2005 | FJ886887 | FJ886961 | FJ887035 |
