# Supplementary material for: Host-Plant Species Conservatism and Ecology of a Parasitoid Fig Wasp Genus (Chalcidoidea; Sycoryctinae; Arachonia)
Source: PLoS One. 2012 Sep 10;7(9):e44804. doi: 10.1371/journal.pone.0044804 (PMC3438170; doi:10.1371/journal.pone.0044804)
Supplement: Table S2 — An inventory of potential host fig wasp species specialising on the Ficus species from which Arachonia were collected in this study. The literature suggests that Sycoryctinae target pollinators mostly and other non-pollinator species only infrequently [82], [83]. There is no hard evidence supporting this and we suspect that sycoryctines equally target the sycoecines and otitesellines associated with section Galoglychia. Arachonia species might also be attacking the Sycophaginae belonging to section Sycomorus in addition to the pollinators. The ratio of parasitoid fig wasp genera to other pteromalids and the agaonids is potentially 2∶1 for each Ficus species from which collections were made in Kibale. However, relatively smaller parasitoid population sizes and differences in species diversity might instead be a clue to fundamentally different evolutionary diversification processes [84]. Typically, phytophagous insect species are more abundant than parasitoid species [85], [86] and are also attacked by more than one parasitoid species [87]. These observations are consistent with our records and other fig wasp studies. Compton and colleagues [88] showed that the ratio of pollinator to non-pollinator fig wasp abundance in forest patches in Asia was approximately 3∶1. The Epichrysomallinae comprised between 45% and 75% of all the non-pollinator sub-families with fewer still of the Otitesellinae and Sycoryctinae that were in roughly equal abundance followed by the Sycoecinae and lastly the Sycophaginae. The Epichrysomallinae are gallers of fig seeds and ovules [89] and are parasitised by the Eurytomidae. Weiblen's [83] review shows a food web summary among parasitoids, other fig wasps, and Ficus. The trophic interactions indicate the Agaoninae and Sychophaginae as prey species of the Sycoryctinae, but did not recognise those between the Otitesellinae and Sycoecinae. Infrequent interactions with the Otitesellinae have been observed [89]. (DOC) [file pone.0044804.s008.doc]

**Table S2: An inventory of potential host fig wasp species specialising on the *Ficus* species from which *Arachonia*** were collected in this study.

| HOST FIG | AGAONIDAE | SYCOECINAE | OTITESELLINAE | SYCOPHAGINAE | SYCORYCTINAE |
| --- | --- | --- | --- | --- | --- |
| *Ficus artocarpoides* Warberg | *Courtella hladikae* (Wiebes) | *Crossogaster michaloudi* van Noort; *Seres longicalcar* van Noort. | *Otitesella sp*. | None | *Sycoscapter sp.* A*;* *Sycoscapter sp*. B; *Sycoryctes sp*. A; *Arachonia sp*.; *Watshamiella* *sp.* A; *Watshamiella sp*. B; *Watshamiella* *sp*. C; *Philotrypesis* *sp*. A; *Philotrypesis sp.* B. |
| *Ficus bizanae* Hutch. & Burtt-Davy | *Courtella sp.* | *Crossogaster* *sp*. | *Otitesella sp.* | None | *Sycoscapter sp.*; *Arachonia sp.*; *Philotrypesis sp.* |
| *Ficus bubu* Warberg | *Courtella michaloudi* (Wiebes). | *Seres wardi* van Noort. | *Otitesella sp*. 1; *Otitesella* *sp*. 2 | None | *Sycoscapter sp*.; *Watshamiella sp*. A; *Watshamiella sp.* B; *Sycoryctes sp*. A; *Sycorcytes sp.* B; *Sycoryctes sp*. C; *Sycoryctes sp*. D; *Sycoryctes sp*. E; *Sycoryctes sp*. F; *Arachonia sp*. A; *Arachonia sp*. B. |
| *Ficus chirindensis* Berg | *Courtella malawi* Wiebes. | *Crossogaster sp*. | *Otitesella sp*. 1; *Otitesella* *sp*. 2 | None | *Arachonia Sycoryctes* sp.; Sycoscapter ; *Watshamiella* sp. *Philotrypsesis sp* A; *Philotrypesis sp*. B |
| *Ficus ovata* Vahl | *Courtella hamifera modesta* (Wiebes). | *Seres solweziensis* van Noort. | *Otitesella sp*. | None | *Sycoryctes sp*.; *Sycoscapter sp*.; *Arachonia* *sp*. A; *Arachonia sp*. B; *Watshamiella sp*.; *Philotrypesis sp*. |
| *Ficus polita* Vahl | *Courtella bekiliensis bispinosa* (Wiebes). | *Crossogaster sp.* | *Otitesella sp*. | None | Sycoryctes sp. A; Sycoyctes sp. B; Sycoscapter; Arachonia Watshamiella |
| *Ficus sansibarica macrosperma* Mildbr. and Burret | *Courtella armata* (Wiebes). | *Seres sp*.; *Crossogaster inusitata* van Noort. | *Otitesella sp*. 1; *Otitsella s*p. 2 | None | *Sycoscapter sp.*; *Sycoryctes sp*. A; *Sycorcytes sp*. B; *Arachonia* *sp.*; *Philotrypesis sp.*; *Watshamiella* *sp*. A; *Watshamiella* *sp*. B. |
| *Ficus sansibarica sansibarica* Warberg | *Courtella armata* (Wiebes). | *Seres solweziensis* van Noort. | *Otitesella sp*. 1; *Otitsella sp*. 2 | None | *Sycoscapter*; *Sycoryctes* sp A; *Sycorcytes*sp. B*; Arachonia* ; *Philotrypesis;* *Watshamiella* sp,. A; *Watshamiella* sp B |
| *Ficus sur* Forsk. | *Ceratosolen silvestrianus* Grandi; *Ceratosolen capensis* Grandi. | None | None | *Apocryptophagus sp.* 1; *Apocryptophagus sp*. 2; *Apocryptophagus sp*. 3; *Sycophaga silvestrii* Grandi. | *Arachonia* *sp*. A; *Arachonia sp*. B; *Watshamiella* *sp*. A; *Watshamiella sp*. B; *Watshamiella sp.* C; *Apocrypta* *sp*. A; *Apocrypta sp.* B. |
| *Ficus sycomorus* L. | *Ceratosolen arabicus* Mayr; *Ceratosolen galili* Wiebes. | None | None | *Sycophaga sycomori* (L.); *Apocryptophagus gigas* (Mayr); *Apocryptophagus* *sp*. B; *Eukoebelea sycomori* Wiebes. | *Arachonia* sp. A; *Arachonia* sp. B; *Sycoscapter* sp. *Watshamiella* sp. A *Watshamiella sp*. B; *Apocrypta longitarsus* Mayr; *Apocrypta* *sp*. B |

The literature suggests that Sycoryctinae target pollinators mostly and other non-pollinator species only infrequently [1,2]. There is no hard evidence supporting this and we suspect that sycoryctines equally target the sycoecines and otitesellines associated with section *Galoglychia*. *Arachonia* species might also be attacking the Sycophaginae belonging to section *Sycomorus* in addition to the pollinators. The ratio of parasitoid fig wasp genera to other pteromalids and the agaonids is potentially 2:1 for each *Ficus* species from which collections were made in Kibale. However, relatively smaller parasitoid population sizes and differences in species diversity might instead be a clue to fundamentally different evolutionary diversification processes [3]. Typically, phytophagous insect species are more abundant than parasitoid species [4,5] and are also attacked by more than one parasitoid species [6]. These observations are consistent with our records and other fig wasp studies. Compton and colleagues [7] showed that the ratio of pollinator to non-pollinator fig wasp abundance in forest patches in Asia was approximately 3:1. The Epichrysomallinae comprised between 45% and 75% of all the non-pollinator sub-families with fewer still of the Otitesellinae and Sycoryctinae that were in roughly equal abundance followed by the Sycoecinae and lastly the Sycophaginae. The Epichrysomallinae are gallers of fig seeds and ovules [8] and are parasitised by the Eurytomidae. Weiblen’s [2] review shows a food web summary among parasitoids, other fig wasps, and *Ficus*. The trophic interactions indicate the Agaoninae and Sychophaginae as prey species of the Sycoryctinae, but did not recognise those between the Otitesellinae and Sycoecinae. Infrequent interactions with the Otitesellinae have been observed [8].

1. Cook JM, Segar ST (2010) Speciation in fig wasps. Ecol Entomol 35: 54-66.

2. Weiblen GD (2002) How to be a fig wasp. Annu Rev Entomol 47: 299-330.

3. McLeish MJ, van Noort S, Tolley KA (2010) Parasitoid fig-wasp evolutionary diversification and variation in ecological opportunity. Mol Ecol 19: 1483-1496.

4. Holt RD, Lawton JH (1993) Apparent competition and enemy-free space in insect host-parasitoid communities. Am Nat 142: 623-645.

5. Holt RD, Lawton JH, Polis GA, Martinez ND (1999) Trophic rank and the species-area relationship. Ecology 80: 1495-1504.

6. Hawkins BA (1990) Global patterns of parasitair assemblage size. J Anim Ecol 59: 57-72.

7. Compton SG, Ellwood MDF, Low R, Watson J (2005) Dispersal of fig wasps (Hymenoptera: Chalcidoidae) across primary and logged rainforest in Sabah (Malaysia). Acta Societas Zoologicae Bohemicae 69: 37-48.

8. Compton SG, van Noort S (1992) Southern African fig wasp assemblages: host relationships and resource utilization. P K Ned Akad Wetensc 95: 423-435*.*
