# Supplementary material for: Host-Plant Species Conservatism and Ecology of a Parasitoid Fig Wasp Genus (Chalcidoidea; Sycoryctinae; Arachonia)
Source: PLoS One. 2012 Sep 10;7(9):e44804. doi: 10.1371/journal.pone.0044804 (PMC3438170; doi:10.1371/journal.pone.0044804)
Supplement: Table S3 — Morphological delimitation of Arachonia species collected in this study showing their haplotype affinities. (DOC) [file pone.0044804.s009.doc]

**Table S3: Morphological delimitation of *Arachonia*** species collected in this study showing their haplotype affinities.

| ***Arachonia* species** | **Haplotype affinity** | **Diagnostic characters** |
| --- | --- | --- |
| *Arachonia* species 1 | Ic & Id | Toruli situated on lower ocular line. Ovipositor 4.4X metasomal length; 7.25X body length (with head in prognathous position). Propodeum nucha invagination broad, width to medial longitudinal length of propodeum 13.5:5. Postmarginal vein longer than stigmal (32:25). Body blueish-black. |
| *Arachonia* species 2 | II | Toruli situated on lower ocular line. Ovipositor 5.1X metasomal length; 11.25X body length (with head in prognathous position). Propodeum nucha invagination deep - width to medial longitudinal length of propodeum 12:5. Postmarginal vein subequal in length to stigmal (28:27). Body blueish-black. |
| *Arachonia* species 3 | Ib | Toruli situated below lower ocular line. Head subquadrate, length to width = 0.90X. Ovipositor 9.3X body length, Body green, legs yellow. |
| *Arachonia* species 4 | III | Toruli situated below lower ocular line. MPS on first antennal funicle widely spaced. Ovipositor 8.5X metasomal length; 4.5X body length. Body mostly green except for lower face and mesosoma and legs. |
| *Arachonia* species 5 | Outlier | Toruli situated below lower ocular line. Antennal anelli transverse (3-4X wider than long). Ovipositor 8.4X metasomal length; 4.4X body length (with head in prognathous position). Body yellow, with narrow darker medial stripe dorsally on metasoma. |
| *Arachonia* species 6 | Outlier | Toruli situated below lower ocular line. Antennal anelli subquadrate (first anellus only slightly wider than long). Ovipositor 7.4X metasomal length; 3.85X body length (with head in prognathous position). Body yellowish brown, green refringence dorsally. |
| *Arachonia* species 7 | Ia & IV | Toruli situated below lower ocular line. MPS on first antennal funicle narrowly spaced. Ovipositor 9X metasomal length; 4.9X body length. Body combination of yellow and green (metasoma and parts of dorsal mesosoma green; rest of mesosoma and anterior half of head yellow). |
